# DNCON2_Inter: predicting interchain contacts for homodimeric and homomultimeric protein complexes using multiple sequence alignments of monomers and deep learning

**DOI:** 10.1038/s41598-021-91827-7

**Published:** 2021-06-10

**Authors:** Farhan Quadir, Raj S. Roy, Randal Halfmann, Jianlin Cheng

**Affiliations:** 1 Bioinformatics and Machine Learning (BML) Lab, Department of Electrical Engineering and Computer Science (EECS), University of Missouri-Columbia, Columbia, MO USA; 2 Stowers Institute for Medical Research, Kansas City, MO USA

**Keywords:** Bioinformatics, Protein structure predictions

## Abstract

Deep learning methods that achieved great success in predicting *intrachain* residue-residue contacts have been applied to predict *interchain* contacts between proteins. However, these methods require multiple sequence alignments (MSAs) of a pair of interacting proteins (dimers) as input, which are often difficult to obtain because there are not many known protein complexes available to generate MSAs of sufficient depth for a pair of proteins. In recognizing that multiple sequence alignments of a monomer that forms homomultimers contain the co-evolutionary signals of both intrachain and interchain residue pairs in contact, we applied DNCON2 (a deep learning-based protein intrachain residue-residue contact predictor) to predict both intrachain and interchain contacts for homomultimers using multiple sequence alignment (MSA) and other co-evolutionary features of a single monomer followed by discrimination of interchain and intrachain contacts according to the tertiary structure of the monomer. We name this tool DNCON2_Inter. Allowing true-positive predictions within two residue shifts, the best average precision was obtained for the Top-L/10 predictions of 22.9% for homodimers and 17.0% for higher-order homomultimers. In some instances, especially where interchain contact densities are high, DNCON2_Inter predicted interchain contacts with 100% precision. We also developed Con_Complex, a complex structure reconstruction tool that uses predicted contacts to produce the structure of the complex. Using Con_Complex, we show that the predicted contacts can be used to accurately construct the structure of some complexes. Our experiment demonstrates that monomeric multiple sequence alignments can be used with deep learning to predict interchain contacts of homomeric proteins.

## Introduction

Proteins are one of the most important and heavily studied biological molecules. While most proteins form individual three-dimensional structures, they tend to interact with each other to gain functional properties. In fact, most proteins are symmetrical oligomeric complexes with two or more subunits^1^, and approximately two-thirds of human enzymes are homo-oligomers^2^.

Since the functionality of most proteins heavily depends on oligomerization, and wet laboratory experiments with actual proteins are time-consuming and expensive, there is a need to develop accurate computational tools to make such predictions quickly. Machine learning methods have been developed to facilitate computational modeling of both protein tertiary structures and quaternary structures. However, recent focus has been on the development of computational tools for predicting intrachain (within the same chain) residue-residue contacts and distances to guide tertiary structure modeling ^3,4^. Some of these methods have performed well in the 12th and 13th Critical Assessment of Techniques for Protein Structure Prediction (CASP) competitions^5–14^.

Unlike tertiary structure modeling, most of the tools on protein complexes are developed to classify whether two proteins are interacting or not, with some tools developed exclusively for docking purposes. Some popular docking tools include RosettaDock^15,16^, ZDOCK^17^, ClusPro^18^, etc. RosettaDock is based on the Monte Carlo method and performs minimum energy optimization-based modeling, while both ZDOCK and ClusPro are based on Fast Fourier transformation. ClusPro computes models with low energy scores and ZDOCK aims to achieve a maximum ZDOCK-score. Only a small number of tools were developed to predict interchain contacts leveraging interchain residue-residue co-evolutionary signals embedded in the MSA of a pair of interacting protein homologues (i.e., interlogs)^3,4,19^. Relevant to the present work, Zhou et al.^4^ and Zeng et al.^20^ predicted interchain contacts using a deep learning-based intrachain contact prediction tool (RaptorX-ComplexContact) without training the system for interchain contact prediction. Their work involved generating MSAs using homology-based, phylogeny-based, and genome-based interlog searches, which is similar to the methods employed by Baker^19^ and Marks^3^. Baker^19^ used a pseudo-likelihood-based covariance approach to predict interprotein contacts in bacterial proteins. The method involved the computation of residue-residue coupling strength between all the interacting protein pairs in the MSA based on the GREMLIN model. The coupling strengths were then ranked and used to compute a score, which was used to derive the distance restraints. EVcomplex^3^ used evolutionary couplings (EC) to predict the interface contacts between prokaryotic proteins. Applying EVcouplings^21^ to the paired MSA and using the pseudo-likelihood maximization (PLM) approach, both inter-EC and intra-EC were obtained. The normalized raw reliability score (EVcomplex score) was calculated using the inter-EC portion. The interchain residue pairs were ranked according to the EVcomplex scores, and pairs with scores ≥ 0.8 were considered to be in contact with high confidence. Another approach by Baker^23^, in order to determine protein–protein interactions in *Escherichia coli* and *Mycobacterium tuberculosis*, selected strongly co-evolving interacting protein pairs from the paired MSA of orthologs based on high values of residue-residue mutual information. The selected proteins were further analyzed using direct coupling analysis (DCA) followed by GREMLIN. It was also determined that applying deep learning methods like DeepCov^24^ that was exclusively trained to predict intrachain contacts, did not improve interchain contact prediction of heterodimers when compared to their approach of using DCA followed by GREMLIN. It was further suggested that in order to use such deep learning approaches, the networks should be trained exclusively for interprotein contact prediction.

The works above focused on determining the *heteromeric* interprotein contacts and require MSAs of homologous interlogs as input. However, obtaining MSAs of interlogs of sufficient depth is difficult because there are fewer known protein complexes than known monomers. However, the situation is different for homo-oligomers because their units are the same monomer. Therefore, both intrachain and interchain residue-residue co-evolutionary signals exist within the same MSA of the monomer. That is, both the co-evolution signal between residues i and j within a monomer and that between residue i in the monomer and residue j in its identical partner are mixed in the MSA of the monomer. This phenomenon has been recognized before but has not been leveraged to predict interchain contacts in homo-oligomers^25^.

The objective of this study focuses on predicting the interprotein contact of *homomeric* proteins (chains having similar amino acid sequences) using MSAs of the monomer unit in homo-oligomers. Similar to Zeng et al.^20^ and Zhou et al.^4^, our approach can be applied to prokaryotic and eukaryotic proteins wherever sufficiently deep MSAs are available. Unlike the above approach to generate MSAs, ours directly extracts co-evolutionary features of homomeric proteins from homology-based MSAs constructed without any special genome or phylogeny-based methods. We then directly apply DNCON2^5^, a deep learning method trained to predict intrachain contacts, to these features to predict both intrachain and interchain contacts, which we distinguish according to the tertiary structures of monomers (See Supplementary Section Figure S1 for the DNCON2 deep learning architecture, and Table S1 for the list of features used by DNCON2). This paper focuses on proving this concept, while future work will focus on developing an exclusive deep-learning-based interprotein contact predictor. Since this development is based on DNCON2, we name our method DNCON2_Inter. Interestingly, DeepHomo^22^ , applying a deep residual network to features derived from monomeric MSA, was recently developed to predict homodimeric interprotein (interchain) contacts of C2 symmetry type proteins, further demonstrating that this direction is promising.

Interchain protein contact predictions are not only useful to identify protein–protein interactions but also in the construction of complex structures. Some docking tools use interchain contacts as additional restraints to compute better quaternary structures. Since more accurate interprotein contacts lead to better quality quaternary structures, this research will pave the way to improve de novo interprotein contact prediction and complex structure construction methods.

## Methods

### Dataset preparation

**Figure 1:**
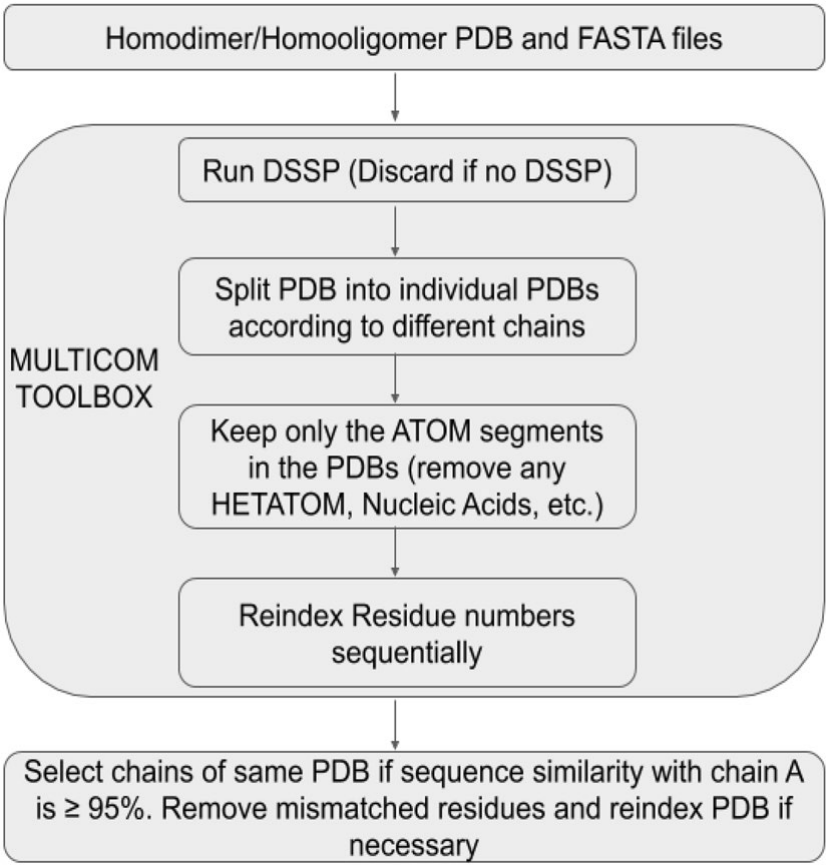
Diagram describing how the input PDB file was pre-processed using the MULTICOM TOOLBOX to clean up the PDB files. If no DSSP is available, the PDB was removed from our list. The individual chains in the multimer PDB were separated into individual files containing the ATOM (x, y, and z coordinates) segments only while discarding all other information. Only chain pairs whose FASTA sequences match 95% or more were kept, and any mismatched residues were removed to ensure homogeneity between chains.

The list of homo-oligomers was obtained from the 3DComplex dataset^26^. We selected our protein dataset after the Protein Data Bank (PDB) files underwent a cascade of filtration steps. We discarded the proteins that had interactions with nucleic acids and cleaned PDB files according to the process described in Fig. 1.

Since PDB files tend to contain more information than necessary, we cleaned them using MULTICOM Toolbox^14,27^, as shown in Fig. 1. DSSP^28^ is applied to generate secondary structure and solvent accessibility information for each PDB file. If DSSP can be generated for a protein, the entire PDB file is split into individual files corresponding to the chains present, keeping only the ATOM (x, y, and z coordinate) portion. The residue numbers are then reindexed to ensure every residue number begins with a '1' and continues without any breaks. If the FASTA sequence similarity between chains is less than 95%, we discard the protein. The individual chain-wise files are further processed to remove the mismatched residue information, and residue numbers were reindexed, if necessary, for homogenizing the residue similarity between chains.

Proteins with more than 30% sequence identity were removed using the "mmseqs"^29^. We also excluded the proteins which did not have any interchain contact (distance between interchain residues was > 6.0 Å). Several proteins that failed with FreeContact^30^, PSICOV^31^, and PSI-BLAST^32^ in the feature generation process of DNCON2 were also excluded. Finally, we obtained a dataset of 8681 homodimeric proteins and 6764 higher-order homo-oligomeric (hereafter, "homomultimeric") proteins for prediction and analysis. The homodimeric and homomultimeric datasets did not overlap and were treated separately.

### Prediction and evaluation of interchain contacts

**Figure 2:**
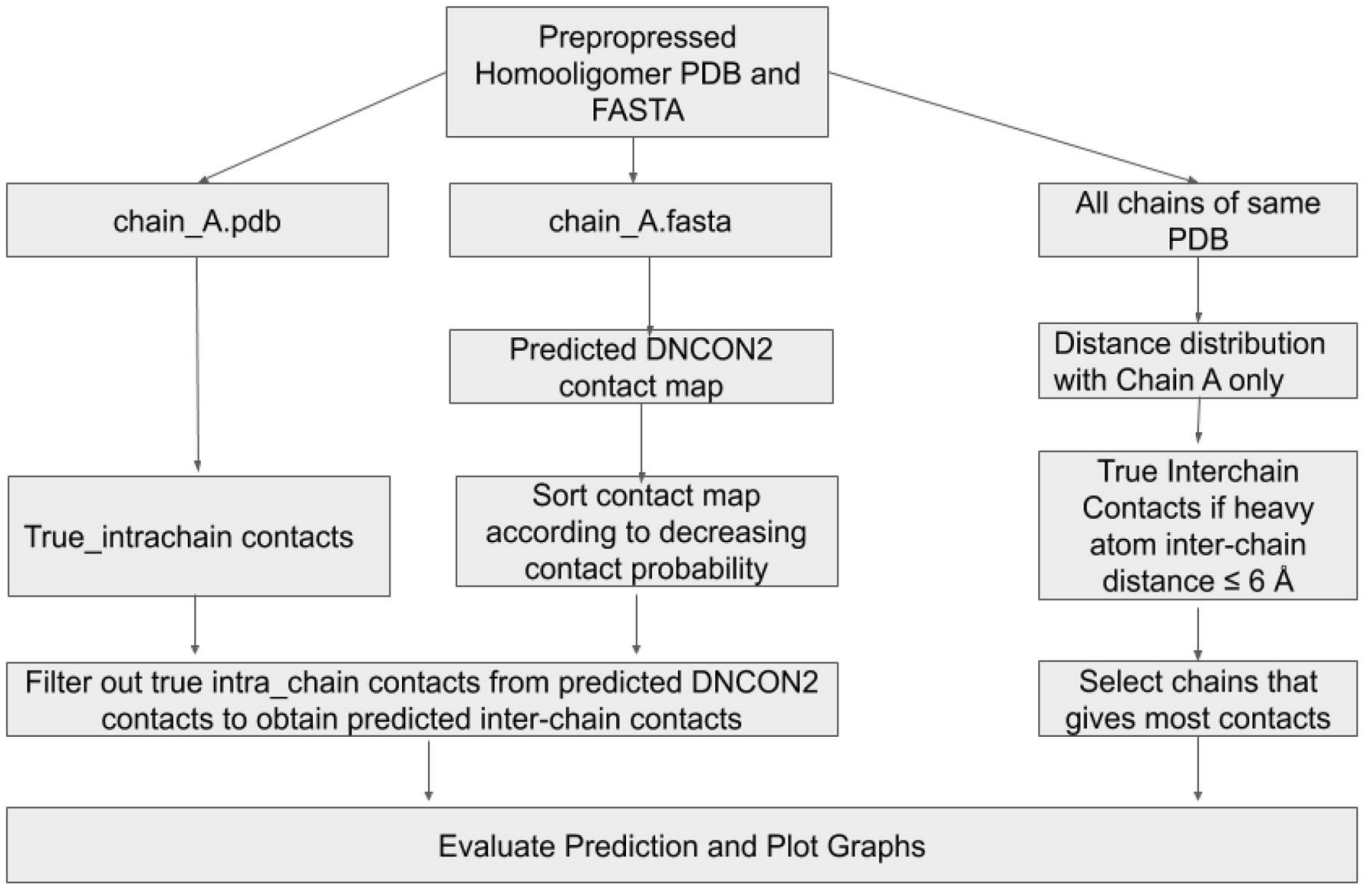
Workflow diagram describing how the pre-processed input PDB and FASTA sequence was used to derive true intrachain contacts, true interchain contacts, predicted interchain contacts, and finally, obtain the evaluation and visualization of the prediction.

Since dimeric proteins contain two chains, the underlying process is relatively simple. Hence, in this study, we only describe predicting the homomultimeric proteins' interchain contacts, which implicitly covers the dimers. As described in Fig. 2, the cleaned chain-wise split PDBs and FASTA sequences were given input to our prediction and evaluation system. The coordinates of chain 'A' (the first chain) were used to compute the intrachain contacts. An *intrachain* contact between two residues i and j is said to exist if the Euclidean distance between the respective C_β_ (C_α_ for glycine) atoms of residues i and j is less than or equal to 8.0 Å^5,14^. If the C_β_ was unavailable, we chose C_α_ instead. We also defined *interchain* contact between chains in a protein if the Euclidean distance between the heavy atoms of the residues in the respective chains is less than or equal to 6.0 Å^3,4,19^. We obtained a pairwise contact list between the first chain (chain A) and the other chains. For example, if a protein contains four chains A, B, C, and D, we pair them as AB, AC, and AD and determine the interchain contacts. We select the pair with the highest number of contacts from this distribution and base the rest of our analysis on this.

To predict the interchain contacts, the FASTA sequence of the first chain (i.e., chain A) was fed into our predictor program- DNCON2- which outputs the predicted intrachain contact map. This predicted contact map was processed to filter out the short-range contacts (contacts whose sequence separation is less than 6). The interchain contacts are obtained as follows:

1. We removed the matching true intrachain contacts from the prediction. Our assumption states that the remaining contacts in the prediction contact map should correspond to some sort of interchain contact.
2. We then computed the precision using the contact map obtained from (1) and the true interchain contacts using InterConEva- an extension of ConEva^33^ tool for interchain contact evaluation. It should be noted that DNCON2 outputs the upper triangle of the prediction matrix since the intrachain contact map is symmetric. However, in interchain prediction, the entire contact matrix needs to be considered since contact between residues i and j of two different chains can be different from contact between residue j and i. This is because, although most homodimeric interactions are symmetric, it is possible to have asymmetric interactions of homodimers within higher order homo-oligomeric complexes. If the final predicted contact map had a contact (x, y) [i.e. between residues x and y], we checked for existence of contact at both positions (x, y) and (y, x) in the true interchain contact map and considered them to be two separate contacts.

We experimented further by applying relaxations (two sets of less strict criteria) to the above parts (1) and (2). We termed them as "relax removal" and "relaxation," respectively. During relax removal, we removed the true intrachain contacts from the predicted contact map as follows:

1. If position (i,j) is a true intrachain contact, and the relax parameter is n (where, n = 0, 1, and 2), then let X = [i − n, i + n] and Y = [j − n, j + n]
2. Remove all contacts (X_p_,Y_q_) from the predicted contact map where X_p_ = {i − n, i − n + 1, …, i + n} and Y_q_ = {j − n, j − n + 1, …, j + n}. This removes (sets to zero) a square matrix of dimension n x n centered at (i,j) from the predicted intrachain contact map.

Relaxation follows a similar approach. If a prediction is found for the position (i,j), we look for contacts within the square matrix of dimension n x n centered at (i,j) in the true interchain contact map. If any nonzero value is found within the n x n square region centered at (i,j) of the true interchain contacts, it is counted as a successfully predicted contact. The value of n is ranged from 0 to 2 since we perform relaxation within two residue shifts.

We also selected one best-case result and reconstructed the complex structure using our recently developed tool Con_Complex, which can reconstruct the quaternary structure of multimers by leveraging the simulated annealing protocol of CNS (Crystallography and NMR System)^34,35^. It uses the monomer PDB file and the predicted inter-protein contacts to reconstruct the homomultimeric complex structure. We compare interchain contact maps using InterConEva, visualize quaternary structures using Chimera^36^, and calculate TMscore using TM-Align^37^. Additionally, we randomly selected 40 homodimer proteins, half of which had zero Top-5 and Top-10 precisions from our experiment, and compared our results to the predictions made by RaptorX-ComplexContact and DeepHomo webserver.

## Results and discussions

### The precision of interchain contact prediction

**Figure 3:**
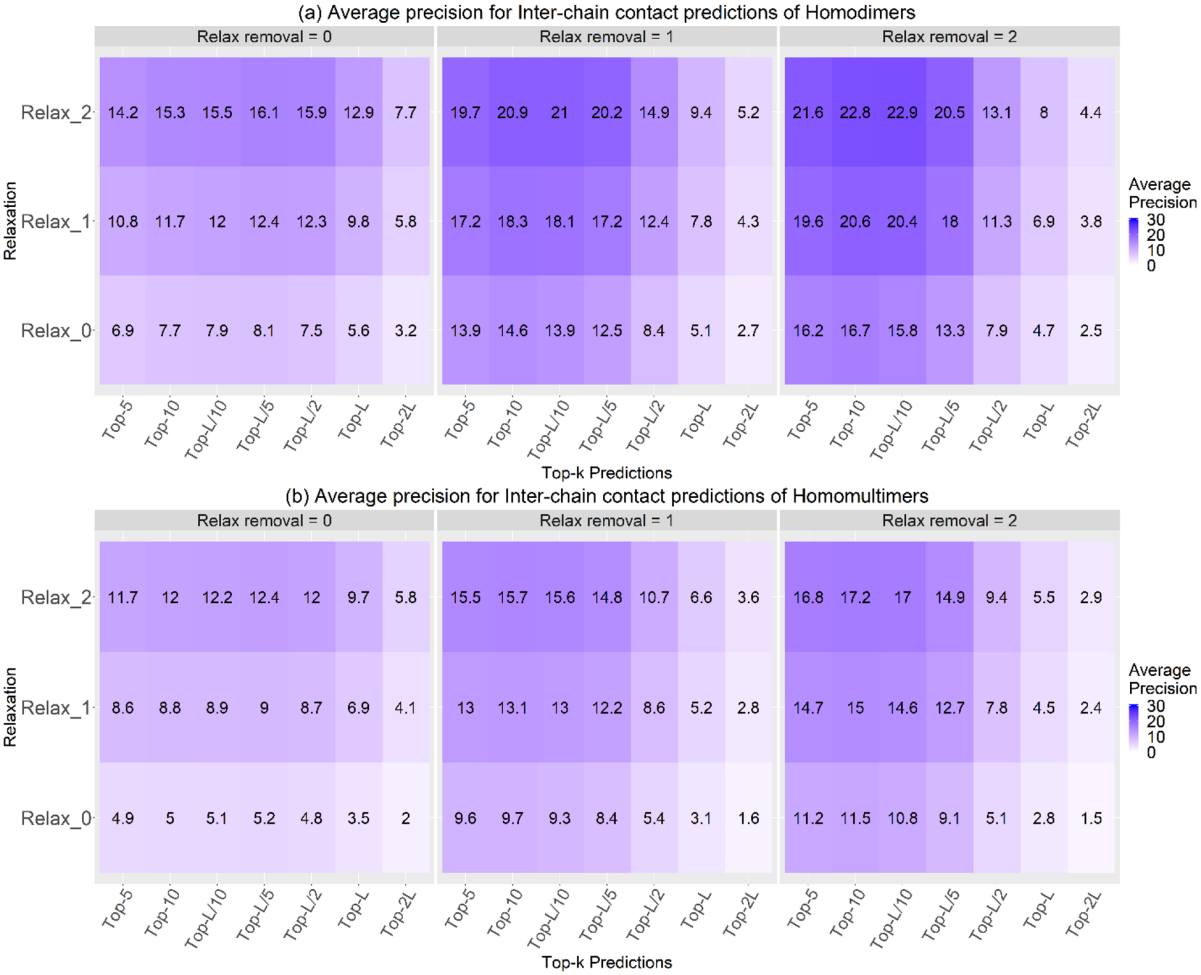
The precision heatmap of interchain contact predictions for the **(a) **homodimers and **(b)** homomultimers for the Top-k predictions where k = 5, 10, L/10, L/5, L/2, L, and 2L. For all categories, as we do more relax removal and relaxation, precision values increase within the respective Top-k categories. Relax removal = 2 and relaxation = 2 shows the best precision of 22.9% for homodimers and 17.0% for homomultimers within the Top-L/10 predictions.

The results from Fig. 3 (Supplementary Section Figure S2 is a line graph of Fig. 3) show that as we perform relax removal and relaxation, the interchain contact prediction precision increases. We obtain a maximum average interchain precision of 22.9% and 17.0% for homodimers and homomultimers, respectively, for the Top-L/10 group with relax removal = 2 and relaxation = 2. When compared with the precision results obtained from random predictions (Supplementary Section Tables S3 and S4), for the Top-L/10 with relax removal = 2 and relaxation = 2, our prediction is 4.1 times greater for homodimers and 3.5 times better for homomultimers. For all groups, increasing relaxation tends to bring about slight increases in precision values. This increase is due to the consideration of true-positive hits over a flexible boundary that enables the system to discover more contacts within a fixed vicinity. However, from Top-L/5 and beyond, precision drops drastically for all the graphs since we are dividing by a bigger number.

**Figure 4:**
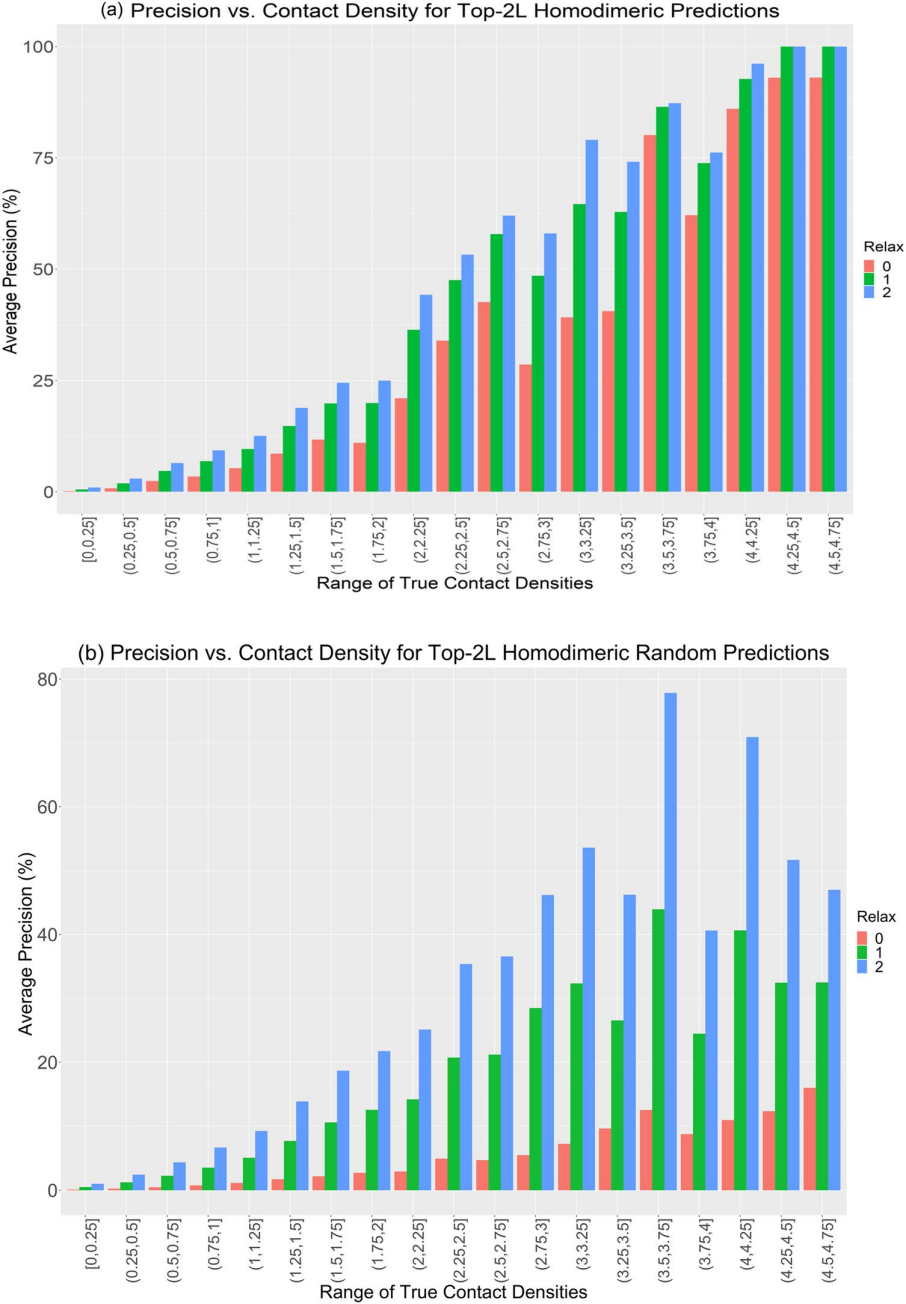
Bar plot depicting the prediction of Top 2L interchain contact predictions changes as contact density varies with no relaxation removal. **(a)** shows the results for DNCON2_Inter predicted contacts and **(b)** shows the results obtained for random prediction. We can see that high contact density leads to high precision. Relaxation has little effect on precision when contact densities are beyond 3.50 for the DNCON2_Inter prediction.

In further analysis, we compare precision against contact density. Contact density is the total number of native contacts per length of the protein^4^. We discuss the contact density and precision of the Top-2L predictions of homodimers without any relax removal. Figure 4 shows that precision increases with increasing contact density for the Top-2L group. We observe that performing relaxation from 0 to 1 increases the precision for contact densities of all ranges. For the DNCON2_Inter predictions (Fig. 4a), precision almost doubles when contact densities are less than 3.50, while, for contact densities beyond 3.50, the precision increases slightly. Performing relaxation from 1 to 2 increases precision but lesser than what is observed when relaxing from 0 to 1. Relaxation from 1 to 2 affects precision values less for contact densities ≥ 3.50. For the case of random predictions (Fig. 4b), similar to the case of DNCON2_Inter, the trend in precision increases until it reaches a contact density of 3.75. In the entire graph, unlike the case for DNCON2_Inter prediction where 100% precision is observed (relaxation 2; contact density 4.25 to 4.75), random prediction achieves the maximum average precision of 77.8% when relaxation is 2 and contact density is between 3.50 and 3.75. For contact densities beyond 3.75, the precisions for random prediction decreases, while DNCON2_Inter predicts contacts with high precisions for proteins with high contact densities. Increasing the relaxation value has a greater impact of increasing precision for random predictions. For DNCON2_Inter, proteins with high contact density get predicted better than proteins with low contact density. It should be noted that most proteins have very sparse interchain contacts and thus have low contact densities. Also, the number of proteins with high contact densities is significantly less (See Supplementary Section Table S2), so these proteins play little role in the overall precision. (Detailed explanation of how relax removal and relaxation effects the precision is described in Supplementary Sect. 10.0. Also, the variation of precision at different contact density thresholds for top predictions for both DNCON2_Inter and random predictions is shown in the heatmaps in Supplementary Sect. 11.0 Figure S8).

### Case study of one of the best predictions

We investigate the results of one of the best predictions PDB code: 1A64. Details are available in the Supplementary Sect. 7.0 (Tables S9 and Figures S3 and S4, respectively).

**Figure 5:**
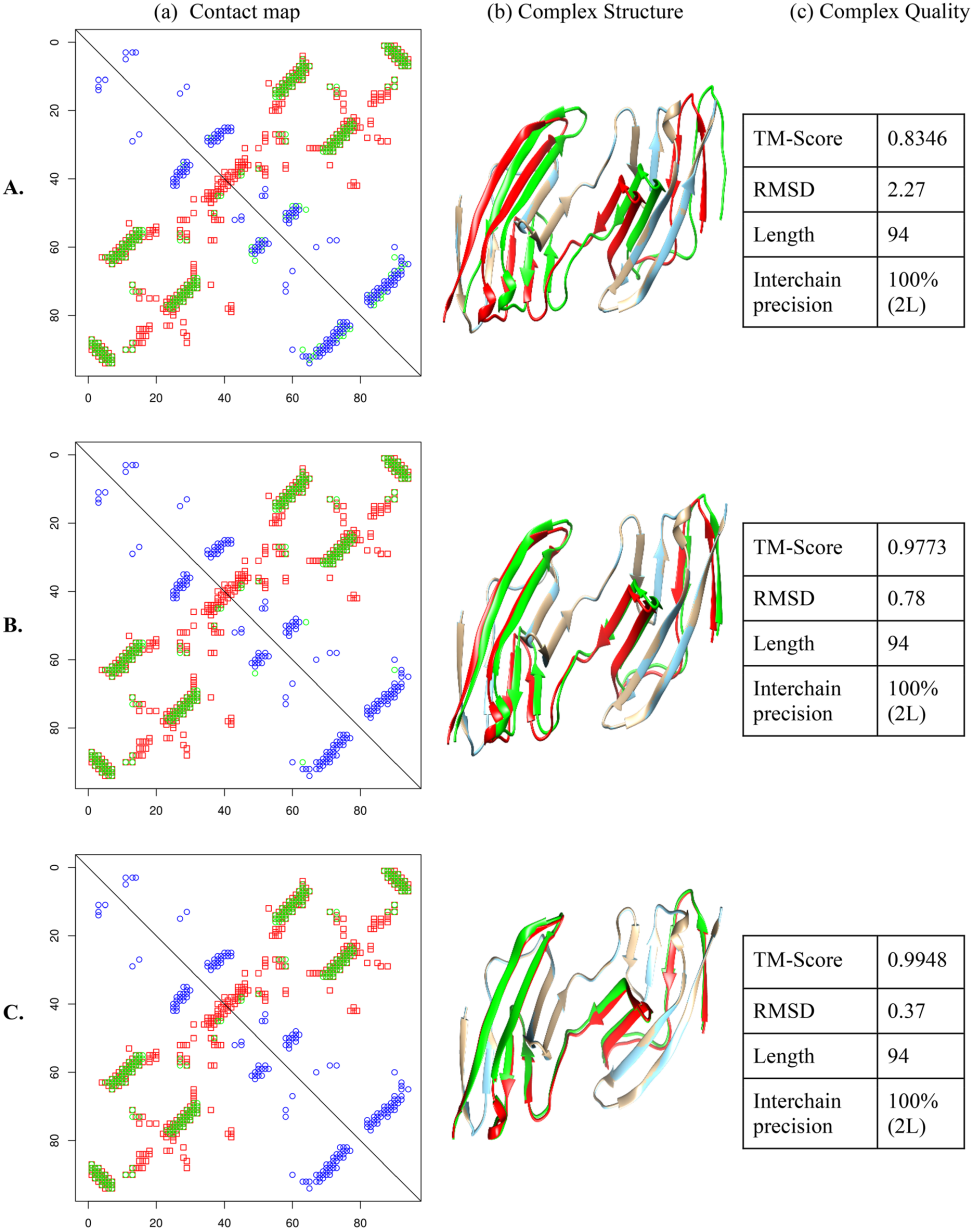
Rows A., B., and C. correspond to relax removal values 0, 1, and 2, respectively. Columns **(a)** show the contact maps, **(b)** shows the complex structure comparison, and **(c)** shows the quality of the complex structures for relevant relax removals A., B., and C. The contact map comparisons are between true intrachain (blue), predicted interchain (green), and true interchain (red) contacts for 1A64. The green dots that overlap with the red dots are correct interchain contact predictions. These contacts were used to reconstruct the complex structure using Con_Complex. **(b)** shows the comparison between true homodimer structure and the structure derived from Con_Complex. (Golden: original chain A; Cyan: reconstructed chain A; red: original chain B; green: reconstructed chain B) The TM-score and RMSDs were obtained using TM-Align and shown in column **(c)**.

Although DNCON2 predicted intrachain contacts for this target with relatively low precision, all the interchain contacts were correctly predicted (Table S9 Supplementary Section), which is also confirmed visually by the bulk overlapping of the green and red dots, as seen in Fig. 5a and Figure S3 (Supplementary Section). As we perform relax removal (A to B to C), the mispredicted green contacts in the contact maps become sparse due to removing the false-positive (assumed to be intrachain) contacts. The green dots that overlap with the red dots are correct interchain contact predictions. Using these contact maps, we use Con_Complex to create quaternary structures and visualize them using Chimera^36^ (Fig. 5b and Supplementary Section Figure S4). Finally, we evaluate the resulting structures' accuracy using TMAlign^37^. In Fig. 5c, our results indicate a very close resemblance to the actual structures with almost perfect TM-scores for the relax removed contacts since intrachain contacts are almost eliminated, leaving behind only true-positive interchain contacts. (Results for another case study on the protein 1IHR can be found in Supplementary Sect. 8.0).

### Comparison between DNCON2_Inter, DeepHomo, and RaptorX-ComplexContact

In our final analysis, we randomly selected 40 homodimer targets, half of which had zero Top-5 and Top-10 precisions from our interchain contact prediction mechanism. We submitted these targets for interchain contact prediction to the RaptorX-ComplexContact webserver and the DeepHomo webserver. For the case of ComplexContact, we apply the exact mechanism of removing true intrachain contacts from the predictions in order to perform a fair comparison with our method. The results from Supplementary Section Table S5 show the details that DNCON2_Inter is approximately 2.7 times better in predicting the Top-L/10 contacts when both relax removal and relaxation are 2. ComplexContact also outputs the intrachain contact map. From this, we similarly removed the true intrachain contacts and re-evaluated the results (details in Supplementary Section Table S6). When comparing the predictions made by the intrachain portion of ComplexContact (ComplexContact_Intra), ComplexContact_Intra performs better than the output of the interchain portion and shows the best results when relax removal is 1 and relaxation is 2. But when compared to DNCON2_Inter, DNCON2_Inter is almost 1.6 times better than ComplexContact_Intra when comparing the Top-L/10 contacts at relax removal = 1 and relaxation = 2.

As DeepHomo directly predicts homodimeric interchain contacts of C2 symmetry, we also compared the performance of DNCON2_Inter with DeepHomo without any relax removal (Summplementary Section Table S7), and DeepHomo outperformed DNCON2_Inter by 1.4 times for the Top-L/10 contacts when relaxation is 2. But, since relax removal of true intrachain contacts is a novelty of DNCON2_Inter, we also compared the results of DeepHomo with the results obtained by DNCON2_Inter at relax removal of 2 units, and the results are available in Supplementary Section Table S8. This increased the precisions of DNCON2_Inter by almost 10% for the Top-L/10 predicted contacts, and DeepHomo still outperformed DNCON2_Inter by about 1.2 times. The better performance achieved by DeepHomo is due to its use of a deep learning network trained exclusively to predict interchain contacts in homodimers from MSAs of monomers, which is expected to be more accurate than DNCON2_Inter that leverages an existing deep network that predicts both intra-chain and inter-chain contacts.

Table 1 shows a comparison of the best results obtained by ComplexContact, ComplexContact_Intra, DeepHomo, and DNCON2_Inter on the randomly selected homodimers at relaxation of 2. It is evident from Table 1 that, for the selected homodimers, DeepHomo outperforms all other methods. DNCON2_Inter outperforms ComplexContact by almost three times for the Top-10 selected contacts. Since ComplexContact bases its MSA on genome-based and phylogeny-based interlogs, it is more suited for heteromeric contact prediction. In addition to DeepHomo’s advantage of training one deep network to exclusively to predict interchain contacts of homodimers, it should also be noted that ten of the selected homodimers occurred in the training data of DeepHomo, which could also result in its performance being significantly better than the other methods. And it is worth noting that one limitation of this study is the small number of proteins used to compare the different methods and study the complex structure construction. The use of a larger dataset in the future may yield better comparisons.

**Table 1:** Comparison between the best precisions (%) of interchain contacts predicted by ComplexContact, ComplexContact_Intra, DeepHomo, and DNCON2_Inter on 40 randomly sampled proteins from the homodimer dataset.

Best precision (%)					
Method	Relax removal	Relaxation	Top-5	Top-10	Top-L/10
ComplexContact—true intra	2	2	18.00	20.00	21.61
ComplexContact_Intra—true intra	1	2	31.50	31.50	35.55
DeepHomo	N/A	2	68.29	67.32	68.02
DNCON2_Inter	2	2	54.15	58.05	57.45

Half of the samples had zero Top-5 and Top-10 precisions as predicted by DNCON2_Inter. For the ComplexContact and ComplexContact_Intra, the evaluation is done after removing the true contacts from the predicted contacts at relax removals 0, 1, and 2. The best precisions for all methods are obtained for relaxation = 2.

## Conclusion, limitation, and future work

This study demonstrates that DNCON2, a deep learning-based intrachain contact predictor, can successfully be used to predict interchain residue-residue contacts for homodimeric and homomultimeric protein complexes from the multiple sequence alignment of a monomer. Although the precision of the predictor is not high on average due to it being trained mainly for intrachain contact prediction, the prediction accuracy is still much higher than a random predictor. In some cases, our approach predicted more interchain contacts than intrachain contacts with very high precision. The results provide good evidence that deep learning tools can be used to train for such a task using co-evolutionary features obtained directly from homology-based MSA of a monomer. Unlike the previous works of Hopf et al.^3^, Ovchinnikov et al.^19^, Zhou et al.^4^, and Zeng et al.^20^, which requires hard-to-obtain MSAs of interlogs as input, our approach can be readily applied to any homodimer and homomultimer. Moreover, the quality of the interchain contacts directly influences complex structure construction. Also, as shown for the cases studied (1A64 and 1IHR), removing false-positive contacts through relax removal can significantly increase the TM-score of the constructed 3D structures of the complexes. Our results conclude that relax removal = 2 and relaxation = 2 give us the best precision, especially for Top-L/10 contacts.

Since contact densities of most complexes are ≤ 1.00, determining how many good contacts are required to produce a good structure of the complex and how false positive contacts play a role remains a challenge and is of future interest. In general, because there are generally fewer interchain contacts than intrachain contacts in homodimers, it is likely that the number of interchain contact predictions required to build quaternary structures of homodimers is smaller than the number of intrachain contact predictions required to build tertiary structures. From our study on limited cases, we can suggest that since the highest precision is obtained for the Top-L/10 predicted contacts, we can start with using the Top-L/10 contacts to build the models of complexes. The number can be adjusted according to specific interactions between two chains. For high-quality modeling of complexes with high contact densities, more contacts may be needed. It is, thus, a future scope for us to explore.

As DNCON2 has been trained for intrachain contact prediction, it has not reached the best performance at predicting interchain contacts. Thus, our results encourage us to develop more advanced complex deep learning architectures specific to predicting interchain contacts of homodimers and homomultimers in the future as shown in DeepHomo. Moreover, large datasets should be used to train and benchmark prediction methods.

## Supplementary Information

Supplementary Information.

## Data Availability

DNCON2_Inter is available to be downloaded from github using the following link: https://github.com/jianlin-cheng/DNCON2_Inter.
