# Supplementary material for: DNCON2_Inter: predicting interchain contacts for homodimeric and homomultimeric protein complexes using multiple sequence alignments of monomers and deep learning
Source: Sci Rep. 2021 Jun 10;11:12295. doi: 10.1038/s41598-021-91827-7 (PMC8192766; doi:10.1038/s41598-021-91827-7)
Supplement: Supplementary file 1 — Supplementary Information. [file 41598_2021_91827_MOESM1_ESM.docx]

**Supplementary Section**

**DNCON2_Inter: Predicting interchain contacts for homodimeric and homomultimeric protein complexes using multiple sequence alignments of monomers and deep learning**

Farhan Quadir^1^, Raj Roy^1^, Randal Halfmann^2^, Jianlin Cheng^1,*^

^1^Bioinformatics and Machine Learning (BML) Lab, Department of Electrical Engineering and Computer Science (EECS), University of Missouri- Columbia, Missouri, USA

^2^Stowers Institute for Medical Research, Kansas City, Missouri, USA

* Corresponding author: [chengji@missouri.edu](mailto:chengji@missouri.edu)

**1.0 DNCON2 Deep Learning Network Architecture for Predicting Intra-chain contacts**


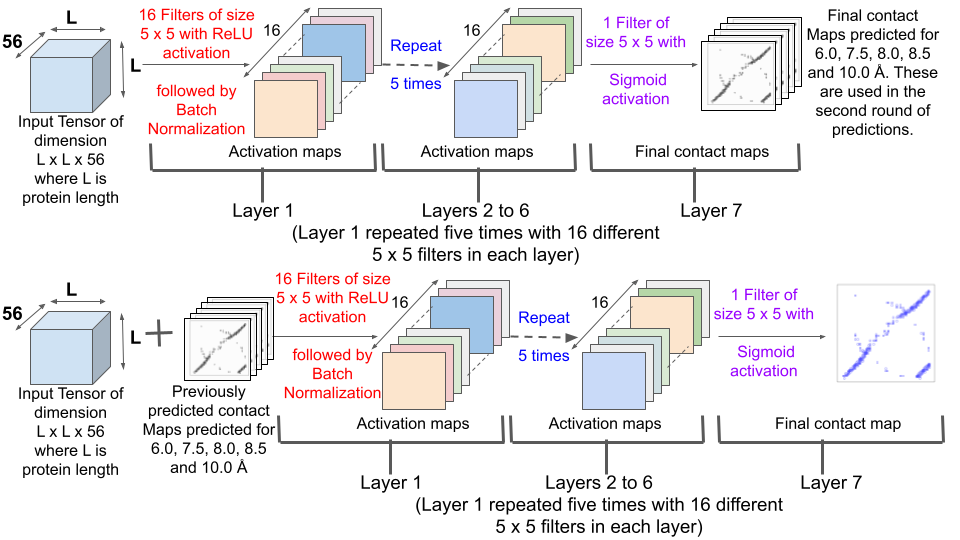


**Figure S1:** The 2D deep convolutional neural network (CNN) architecture of DNCON2. The input is an L x L x 56 tensor (L: length of the protein), and 56 is the number of channels. (Top) The input is passed through a CNN where the first layer consists of 16 different 5 x 5 filters, activated using the Rectified Linear Unit (ReLU) followed by batch normalization. The output of this block is fed into a similar block but with different filters. This is repeated for six layers. The final output layer consists of one 5 x 5 filter, followed by sigmoid activation to predict the L x L contact map. Five different CNNs of similar architecture were trained to predict contacts at thresholds 6.0, 7.5, 8.0, 8.5, and 10.0 Å. (Bottom). These contact maps are then concatenated to the initial input tensor and then trained using a similar network to predict the final contact map at 8.0 Å. The loss function used is binary cross-entropy with Nesterov Adam (nadam) optimizer.

**2.0 DNCON2 features**

**Table S1.** List of features used by DNCON2. Shannon entropy, CCMPred, FreeContact, PSICOV, Mean Contact Potential, Normalized Mutual Information, and Mutual information are co-evolution-based features derived from multiple sequence alignment (MSA).

| **Feature** | **Dimensions** | **Features** | **Channels after augmentation** |
| --- | --- | --- | --- |
| Log of sequence length | Scalar | 1 | 1 |
| Log of number of sequences in the alignment | Scalar | 1 | 1 |
| Log of the number of effective number of sequences in the alignment | Scalar | 1 | 1 |
| The ratio of number of 'buried' residues and length of the protein | 1D | 1 | 1 |
| The ratio of number of 'beta-strand' residues and length of the protein | 1D | 1 | 1 |
| Ratio of number of 'helical' residues and length of the protein | 1D | 1 | 1 |
| Atchley factors normalized by sigmoid function | 1D | 5 | 10 |
| Binary predictions for helix, coil, and strand residues by SCRATCH | 1D | 3 | 6 |
| Solvent accessibility predicted by SCRATCH | 1D | 1 | 2 |
| Position Specific Scoring Matrix (PSSM) | 1D | 1 | 2 |
| PSSM Sums (divided by 100) | 1D | 1 | 2 |
| PSSM sum cosines | 1D | 1 | 2 |
| Ratio of sequence separation and length of protein | 2D | 1 | 1 |
| Flag for sequence separation between 23 and 28 | 2D | 1 | 1 |
| Flag for sequence separation between 28 and 38 | 2D | 1 | 1 |
| Flag for sequence separation between 38 and 48 | 2D | 1 | 1 |
| Flag for sequence separation 48+ | 2D | 1 | 1 |
| Probabilities of PSIPRED predictions for helix, coil, and strand residues | 1D | 3 | 6 |
| Probabilities of PSISOLV predictions for solvent accessibility | 1D | 1 | 2 |
| Pre-computed statistical potentials | 2D | 6 | 6 |
| Shannon entropy sum of the alignment columns | 2D | 1 | 1 |
| CCMpred co-evolution prediction from MSA | 2D | 1 | 1 |
| FreeContact co-evolution prediction from MSA | 2D | 1 | 1 |
| PSICOV co-evolution prediction from MSA | 2D | 1 | 1 |
| Mean contact potential from MSA | 2D | 1 | 1 |
| Normalized mutual information from MSA | 2D | 1 | 1 |
| Mutual information from MSA | 2D | 1 | 1 |
| Total |  | 40 | 56 |

**3.0 Table for Contact Density distribution of proteins**

**Table S2:** Table showing the contact density distribution of homodimers and homomultimers.

| **Number of proteins** | | |
| --- | --- | --- |
| **Contact Density Range** | **Homodimers** | **Homomultimers** |
| 0.00-0.25 | 1291 | 1480 |
| 0.25-0.50 | 2591 | 2053 |
| 0.50-0.75 | 2085 | 1616 |
| 0.75-1.00 | 1302 | 871 |
| 1.00-1.25 | 561 | 392 |
| 1.25-1.50 | 341 | 179 |
| 1.50-1.75 | 163 | 77 |
| 1.75-2.00 | 130 | 37 |
| 2.00-2.25 | 80 | 22 |
| 2.25-2.50 | 43 | 16 |
| 2.50-2.75 | 35 | 15 |
| 2.75-3.00 | 24 | 2 |
| 3.00-3.25 | 16 | 1 |
| 3.25-3.50 | 4 | 1 |
| 3.50-3.75 | 4 | 1 |
| 3.75-4.00 | 2 | 0 |
| 4.00-4.25 | 5 | 0 |
| 4.25-4.50 | 3 | 1 |
| 4.50-4.75 | 1 | 0 |

**4.0 Random Prediction Precision**

**Table S3:** Table showing the precision of the random prediction of homodimers for different relaxation removal and relaxation values.

| Precision (%) | | | | | | | | |
| --- | --- | --- | --- | --- | --- | --- | --- | --- |
| Relax Removal | Relaxation | Top-5 | Top-10 | Top-L/10 | Top-L/5 | Top-L/2 | Top-L | Top-2L |
| 0 | 0 | 0.69 | 0.72 | 0.69 | 0.69 | 0.68 | 0.67 | 0.65 |
| 0 | 1 | 3.07 | 3.13 | 3.09 | 3.10 | 3.09 | 3.11 | 3.04 |
| 0 | 2 | 5.73 | 5.93 | 5.84 | 5.89 | 5.83 | 5.79 | 5.61 |
| 1 | 0 | 0.66 | 0.71 | 0.70 | 0.70 | 0.69 | 0.67 | 0.59 |
| 1 | 1 | 2.95 | 3.01 | 2.98 | 3.04 | 3.04 | 3.03 | 2.69 |
| 1 | 2 | 5.57 | 5.74 | 5.66 | 5.76 | 5.71 | 5.64 | 4.99 |
| 2 | 0 | 0.70 | 0.73 | 0.70 | 0.73 | 0.69 | 0.68 | 0.54 |
| 2 | 1 | 2.95 | 3.01 | 2.95 | 3.01 | 3.02 | 3.01 | 2.43 |
| 2 | 2 | 5.54 | 5.67 | 5.55 | 5.65 | 5.56 | 5.51 | 4.44 |

**Table S4:** Table showing the precision of the random prediction of homomultimers for different relaxation removal and relaxation values.

| Precision (%) | | | | | | | | |
| --- | --- | --- | --- | --- | --- | --- | --- | --- |
| Relax Removal | Relaxation | Top-5 | Top-10 | Top-L/10 | Top-L/5 | Top-L/2 | Top-L | Top-2L |
| 0 | 0 | 0.52 | 0.56 | 0.59 | 0.61 | 0.61 | 0.60 | 0.58 |
| 0 | 1 | 2.67 | 2.74 | 2.77 | 2.76 | 2.74 | 2.70 | 2.63 |
| 0 | 2 | 4.92 | 5.07 | 5.08 | 5.12 | 5.11 | 5.10 | 4.94 |
| 1 | 0 | 0.52 | 0.56 | 0.58 | 0.59 | 0.60 | 0.59 | 0.52 |
| 1 | 1 | 2.59 | 2.66 | 2.68 | 2.70 | 2.66 | 2.65 | 2.31 |
| 1 | 2 | 4.92 | 4.98 | 4.96 | 4.98 | 4.96 | 4.97 | 4.33 |
| 2 | 0 | 0.49 | 0.54 | 0.56 | 0.57 | 0.59 | 0.59 | 0.46 |
| 2 | 1 | 2.52 | 2.60 | 2.63 | 2.63 | 2.61 | 2.61 | 2.05 |
| 2 | 2 | 4.73 | 4.83 | 4.86 | 4.82 | 4.84 | 4.83 | 3.79 |

**5.0 ComplexContact vs DNCON2_Inter vs DeepHomo**

**Table S5:** A comparison between the precisions (%) of the interchain contacts predicted by ComplexContact with the true intrachain contacts removed, and DNCON2_Inter on 40 random proteins sampled from the homodimer dataset. Half of the samples had zero Top-5 and Top-10 precisions as predicted by DNCON2_Inter:

|  | ComplexContact – True intrachain (%) | | | | | | | DNCON2_Inter (%) | | | | | |
| --- | --- | --- | --- | --- | --- | --- | --- | --- | --- | --- | --- | --- | --- |
| Relaxation Removal | Relax | Top- 5 | Top-10 | Top-L/10 | Top-L/5 | Top-L/2 | Top- L | Top- 5 | Top-10 | Top-L/10 | Top-L/5 | Top-L/2 | Top- L |
| 0 | 0 | 6.00 | 7.50 | 7.44 | 7.91 | 8.67 | 8.13 | 35.61 | 36.59 | 36.22 | 33.32 | 27.39 | 18.82 |
| 0 | 1 | 10.50 | 13.50 | 14.40 | 14.48 | 15.91 | 16.39 | 40.98 | 44.15 | 42.59 | 40.39 | 35.72 | 26.20 |
| 0 | 2 | 15.50 | 17.50 | 20.37 | 19.37 | 22.17 | 22.65 | 46.83 | 49.02 | 47.67 | 44.33 | 41.04 | 30.44 |
| 1 | 0 | 7.50 | 8.00 | 8.58 | 10.70 | 10.73 | 8.68 | 48.78 | 51.46 | 49.36 | 42.64 | 28.65 | 17.03 |
| 1 | 1 | 11.00 | 13.50 | 13.77 | 15.74 | 19.12 | 16.82 | 52.68 | 56.59 | 54.16 | 49.88 | 36.61 | 22.42 |
| 1 | 2 | 15.00 | 19.00 | 19.49 | 20.66 | 25.68 | 14.19 | 53.66 | 57.56 | 56.10 | 52.26 | 39.31 | 24.53 |
| 2 | 0 | 7.50 | 9.00 | 10.60 | 11.96 | 11.47 | 8.42 | 48.78 | 50.24 | 49.22 | 49.22 | 26.28 | 15.69 |
| 2 | 1 | 12.00 | 14.25 | 16.43 | 17.98 | 20.45 | 16.14 | 53.17 | 56.10 | 55.31 | 49.61 | 33.39 | 20.44 |
| 2 | 2 | 18.00 | 20.00 | 21.61 | 23.99 | 26.92 | 21.84 | **54.15** | **58.05** | **57.45** | **51.16** | **35.47** | **22.06** |

**Table S6:** A comparison between the precisions of the interchain contacts predicted by ComplexContacts intra portion with true intrachain contacts removed, and DNCON2_Inter on 40 random samples from the homodimer dataset after removing the true contacts from the predicted intrachain contacts. Twenty of the forty proteins had Top-5 and Top-10 precisions to be zero after prediction by DNCON2_Inter:

|  |  | ComplexContact_Intra - True_intra | | | | | | | DNCON2_Inter | | | | | | |
| --- | --- | --- | --- | --- | --- | --- | --- | --- | --- | --- | --- | --- | --- | --- | --- |
| Relax  removal | Relax | Top- 5 | Top-10 | Top-L/10 | Top-L/5 | Top-L/2 | Top- L | Top-2L | Top- 5 | Top-10 | Top-L/10 | Top-L/5 | Top-L/2 | Top- L | Top-2L |
| 0 | 0 | 24.50 | 26.00 | 24.98 | 21.84 | 18.15 | 12.29 | 6.34 | 35.61 | 36.59 | 36.22 | 33.32 | 27.39 | 18.82 | 9.74 |
| 0 | 1 | 26.00 | 30.25 | 29.73 | 26.98 | 23.69 | 17.07 | 9.07 | 40.98 | 44.15 | 42.59 | 40.39 | 35.72 | 26.20 | 13.69 |
| 0 | 2 | 30.50 | 35.35 | 35.26 | 32.17 | 28.80 | 21.22 | 11.35 | 46.83 | 49.02 | 47.67 | 44.33 | 41.04 | 30.44 | 16.05 |
| 1 | 0 | 26.50 | 29.00 | 30.33 | 27.18 | 19.19 | 11.36 | 5.68 | 48.78 | 51.46 | 49.36 | 42.64 | 28.65 | 17.03 | 8.56 |
| 1 | 1 | 29.00 | 30.25 | 33.63 | 30.66 | 23.08 | 14.59 | 7.35 | 52.68 | 56.59 | 54.61 | 49.88 | 36.61 | 22.42 | 11.35 |
| **1** | **2** | **31.50** | **31.50** | **35.55** | **32.97** | **25.50** | **16.46** | **8.29** | **53.66** | **57.56** | **56.10** | **52.26** | **39.31** | **24.53** | **12.44** |
| 2 | 0 | 26.50 | 26.00 | 27.57 | 25.84 | 17.91 | 10.23 | 5.12 | 48.78 | 50.24 | 49.22 | 42.29 | 26.28 | 15.69 | 7.87 |
| 2 | 1 | 28.00 | 28.00 | 30.19 | 28.54 | 21.09 | 12.81 | 6.46 | 53.17 | 56.10 | 55.31 | 49.61 | 33.39 | 20.44 | 10.33 |
| 2 | 2 | 28.00 | 31.25 | 32.89 | 30.95 | 22.99 | 14.04 | 7.08 | 54.15 | 58.05 | 57.45 | 51.16 | 35.47 | 22.06 | 11.18 |

**Table S7:** A comparison between the precisions of the interchain contacts predicted by DeepHomo, and DNCON2_Inter on 40 random samples from the homodimer dataset after removing the true contacts from the predicted intrachain contacts. Twenty of the forty proteins had Top-5 and Top-10 precisions to be zero after prediction by DNCON2_Inter:

|  | DeepHomo | | | | | | | DNCON2_Inter | | | | | | |
| --- | --- | --- | --- | --- | --- | --- | --- | --- | --- | --- | --- | --- | --- | --- |
| Relax | Top- 5 | Top-10 | Top-L/10 | Top-L/5 | Top-L/2 | Top- L | Top-2L | Top- 5 | Top-10 | Top-L/10 | Top-L/5 | Top-L/2 | Top- L | Top-2L |
| 0 | 60.00 | 60.24 | 52.41 | 44.60 | 35.37 | 28.68 | 21.14 | 35.61 | 36.59 | 36.22 | 33.32 | 27.39 | 18.82 | 9.74 |
| 1 | 65.37 | 64.88 | 64.60 | 61.69 | 57.47 | 51.09 | 42.88 | 40.98 | 44.15 | 42.59 | 40.39 | 35.72 | 26.20 | 13.69 |
| 2 | **68.29** | **67.32** | **68.02** | **66.21** | **63.09** | **58.29** | **51.83** | **46.83** | **49.02** | **47.67** | **44.33** | **41.04** | **30.44** | **16.05** |

**Table S8:** A comparison between the precisions of the interchain contacts predicted by DeepHomo, and DNCON2_Inter predictions that have been relax removed by 2 units, on 40 random samples from the homodimer dataset after removing the true contacts from the predicted intrachain contacts. Twenty of the forty proteins had Top-5 and Top-10 precisions to be zero after prediction by DNCON2_Inter:

|  | DeepHomo | | | | | | | DNCON2_Inter | | | | | | |
| --- | --- | --- | --- | --- | --- | --- | --- | --- | --- | --- | --- | --- | --- | --- |
| Relax | Top- 5 | Top-10 | Top-L/10 | Top-L/5 | Top-L/2 | Top- L | Top-2L | Top- 5 | Top-10 | Top-L/10 | Top-L/5 | Top-L/2 | Top- L | Top-2L |
| 0 | 60.00 | 60.24 | 52.41 | 44.60 | 35.37 | 28.68 | 21.14 | 48.78 | 50.24 | 49.22 | 42.29 | 26.28 | 15.69 | 7.87 |
| 1 | 65.37 | 64.88 | 64.60 | 61.69 | 57.47 | 51.09 | 42.88 | 53.17 | 56.10 | 55.31 | 49.61 | 33.39 | 20.44 | 10.33 |
| 2 | **68.29** | **67.32** | **68.02** | **66.21** | **63.09** | **58.29** | **51.83** | **54.15** | **58.05** | **57.45** | **51.16** | **35.47** | **22.06** | **11.18** |

**6.0 Line Graph showing how relax removal and relaxation affect top-k precisions**


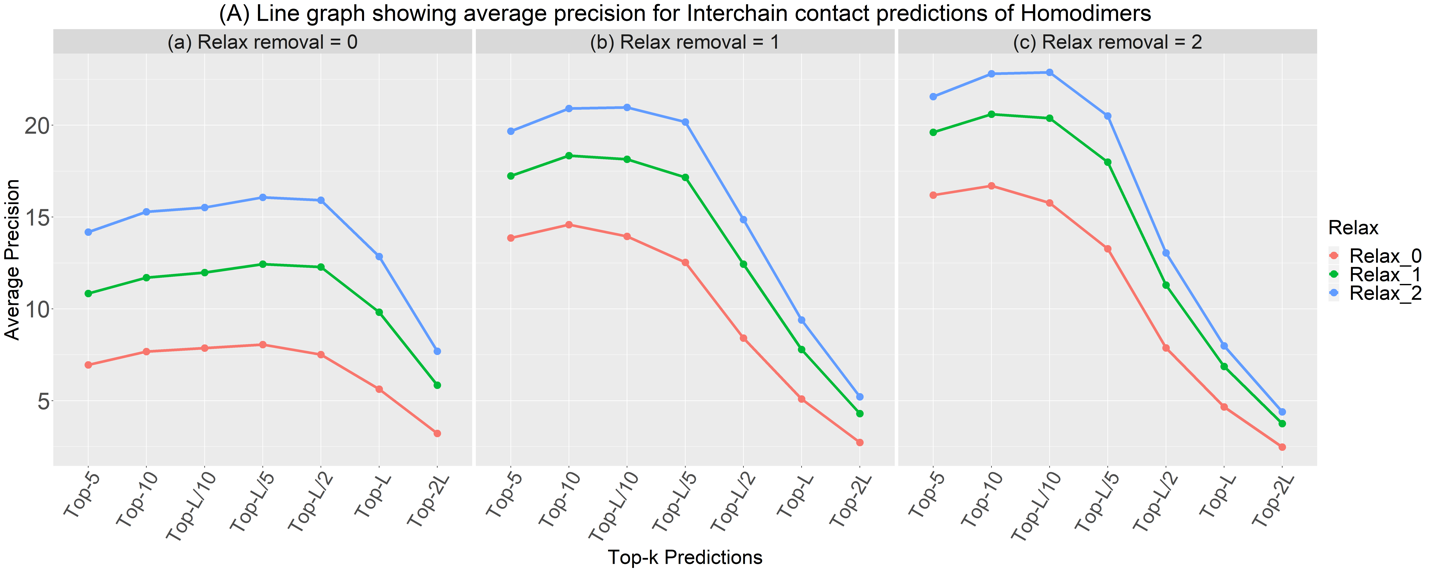


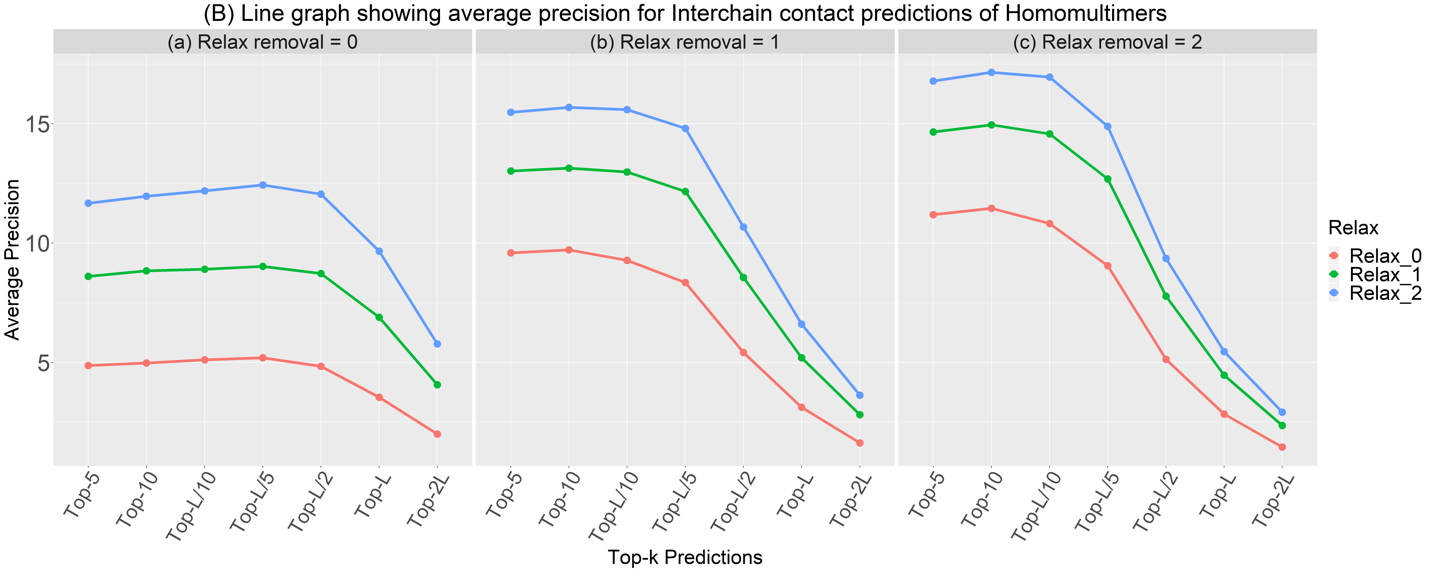


**Figure S2:** The figure shows how relaxation removal and relaxation affect interchain contact prediction precision (A) for homodimers and (B) for homomultimers. For homodimers, (a) there is no relaxation removal. Precision steadily increases for all relaxation thresholds until Top L/5, then sharply decreases. Similar observations are seen for (b) and (c) with maximum precision occurring at Top-10 for (b) and top-L/10 for (c). Increasing relaxation thresholds from 0 to 2 always increases precision.

**7.0 Detailed results for 1A64**

**Table S9**: The precision (%) of intrachain and interchain contact predictions for PDB 1A64. The last three rows correspond to interchain precision at different relaxation removal levels.

| Relax removal | Relaxation | Top-5 | Top-10 | Top-L/10 | Top-L/5 | Top-L/2 | Top-L | Top-2L |
| --- | --- | --- | --- | --- | --- | --- | --- | --- |
| Intrachain Precision | | 0 | 40.0 | 33.33 | 52.63 | 46.81 | 38.30 | 32.45 |
| 0 | 0 | 100 | 100 | 100 | 100 | 100 | 100 | 100 |
| 1 | 0 | 100 | 100 | 100 | 100 | 100 | 100 | 100 |
| 2 | 0 | 100 | 100 | 100 | 100 | 100 | 100 | 100 |

| 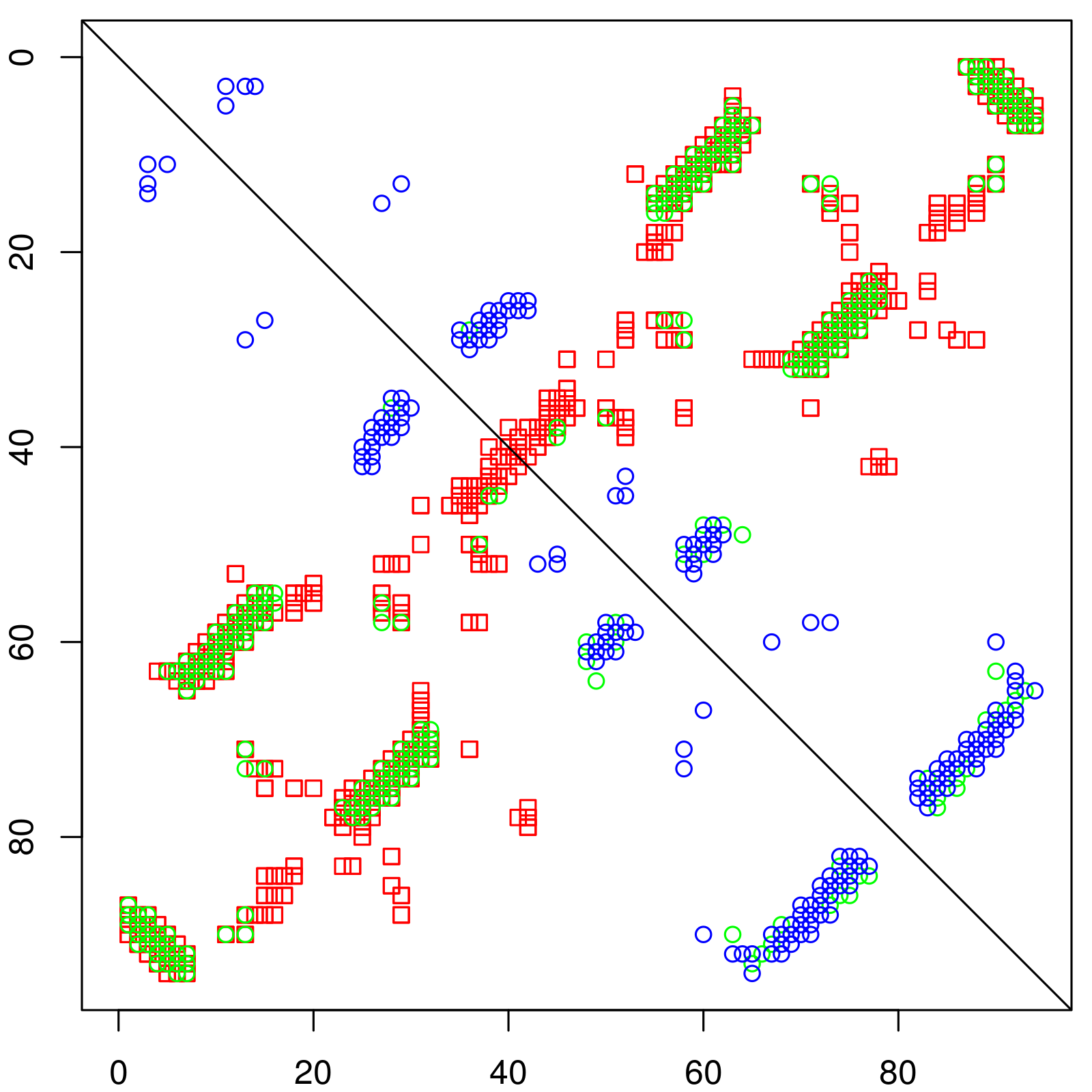(a) No relax removal | 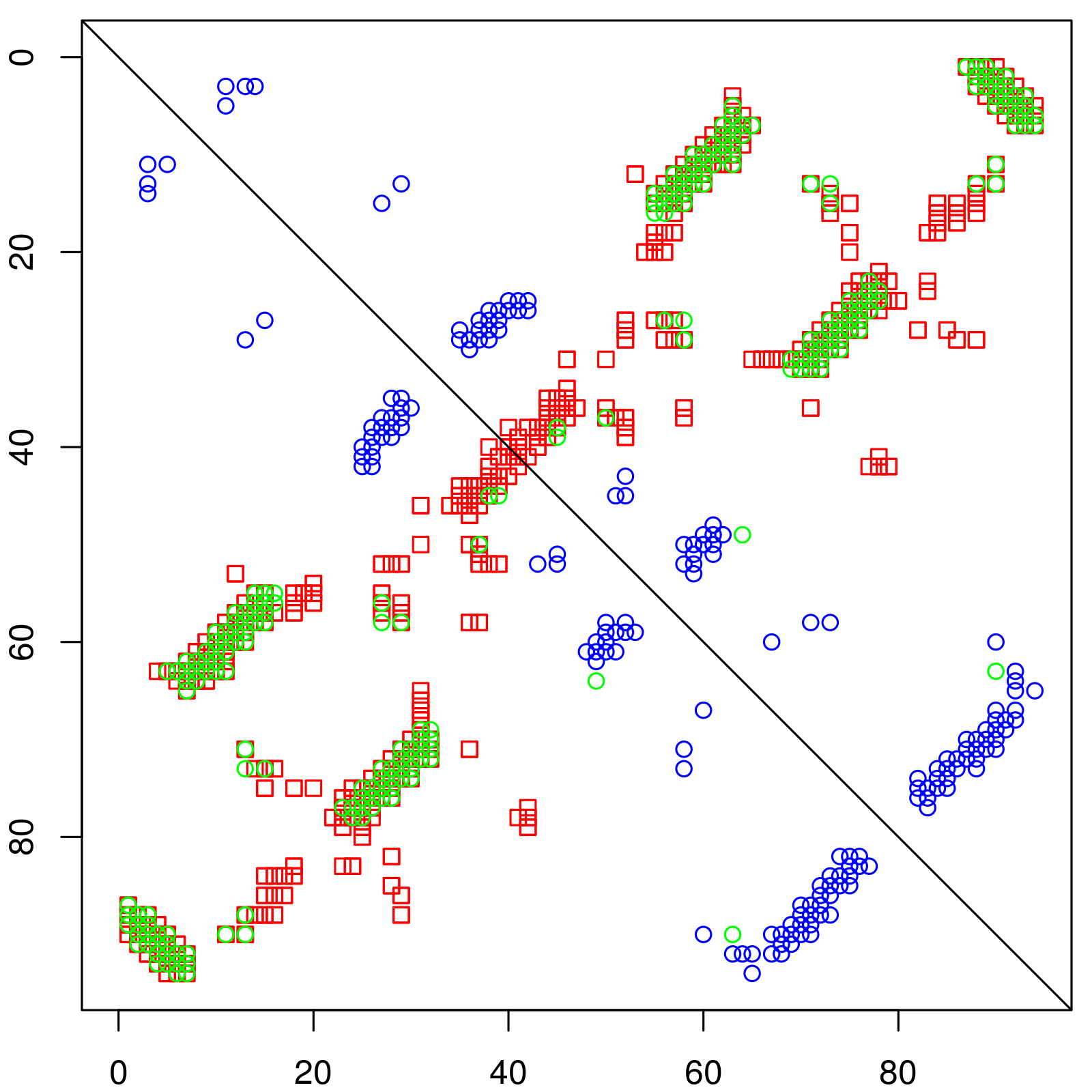(b) Relax removal = 1 | 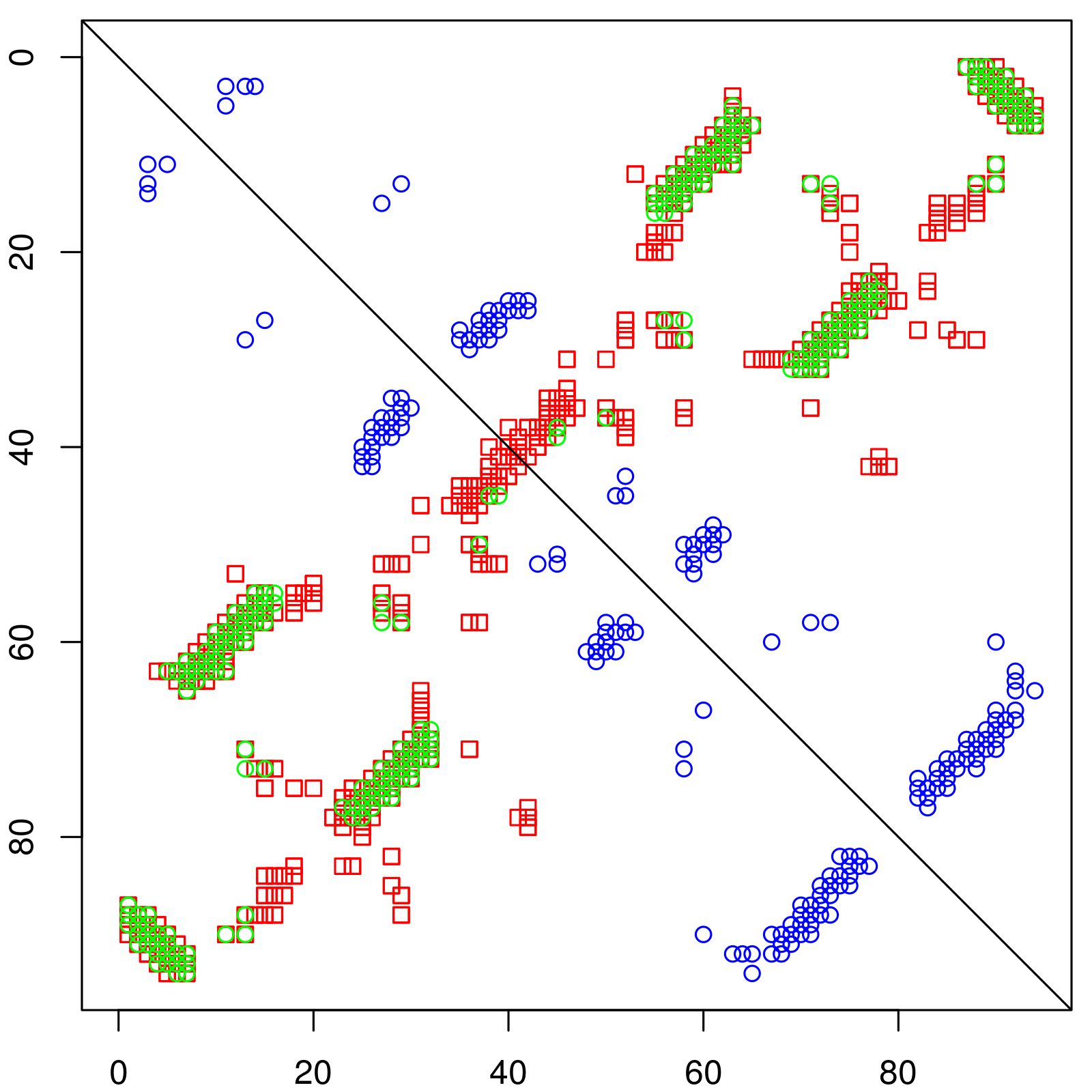(c) Relax removal = 2 |
| --- | --- | --- |

**Figure S3**: Contact map comparison between true intrachain (blue), predicted interchain (green), and true interchain (red) contacts for 1A64. (a) shows more green spots overlapping with the blue dots since no relax removal was done. From (b) to (c), the green contacts become sparse due to removing more predicted contacts assumed to be intrachain. The green dots that overlap with the red dots are correct inter-chain contact predictions.

| 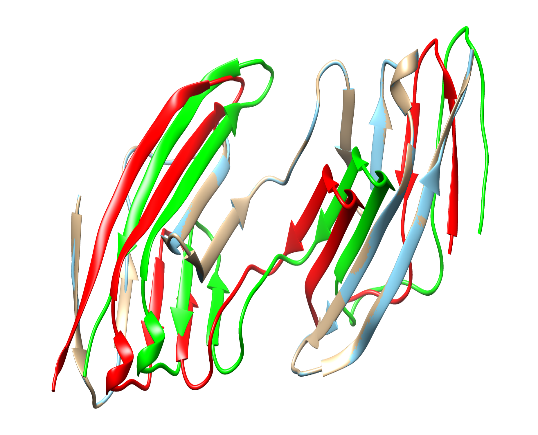  (a) No relax removal | 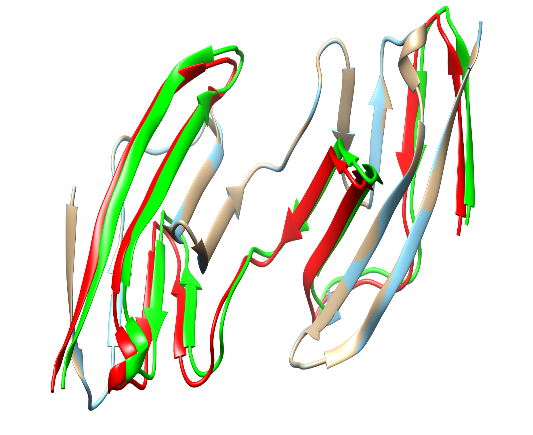  (b) Relax removal = 1 | 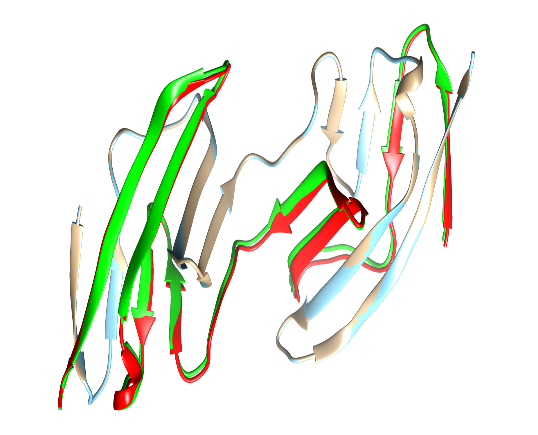  (b) Relax removal = 2 |
| --- | --- | --- |
| \| TM-Score \| 0.83465 \| \| --- \| --- \| \| RMSD \| 2.27 \| \| Length \| 94 \| \| Interchain precision \| 100% (2L) \| | \| TM-Score \| 0.97733 \| \| --- \| --- \| \| RMSD \| 0.78 \| \| Length \| 94 \| \| Interchain precision \| 100% (2L) \| | \| TM-Score \| 0.99481 \| \| --- \| --- \| \| RMSD \| 0.37 \| \| Length \| 94 \| \| Interchain precision \| 100% (2L) \| |

**Figure S4**: Comparison for target 1A64 between its true homodimer structure and the structure derived from our predicted contacts built by CNS (Crystallography and NMR System). (Golden: original chain A; Cyan: reconstructed chain A; red: original chain B; green: reconstructed chain B) The TM-score and RMSDs were obtained using TM-Align. From (a) to (c), as we perform relax removal, we remove more intrachain contacts to obtain a higher proportion of true-positive interchain contacts (as seen in the previous contact map diagram Figure). As a result, the final structures become more accurate, and TM-score increases with decreasing RMSD.

**8.0 Detailed results for 1IHR**

**Table S10:** Table showing the precisions (%) of some top predictions done by our system for PDB 1IHR.

| Relax remove | Relaxation | Top-5 | Top-10 | Top-L/10 | Top-L/5 | Top-L/2 | Top-L | Top-2L |
| --- | --- | --- | --- | --- | --- | --- | --- | --- |
| Intrachain precision | | 40.00 | 50.00 | 28.57 | 57.33 | 45.95 | 29.73 | 18.24 |
| 0 | 0 | 100 | 100 | 100 | 100 | 100 | 100 | 99.32 |
| 1 | 0 | 100 | 100 | 100 | 100 | 100 | 100 | 97.97 |
| 2 | 0 | 100 | 100 | 100 | 100 | 100 | 100 | 89.86 |

| 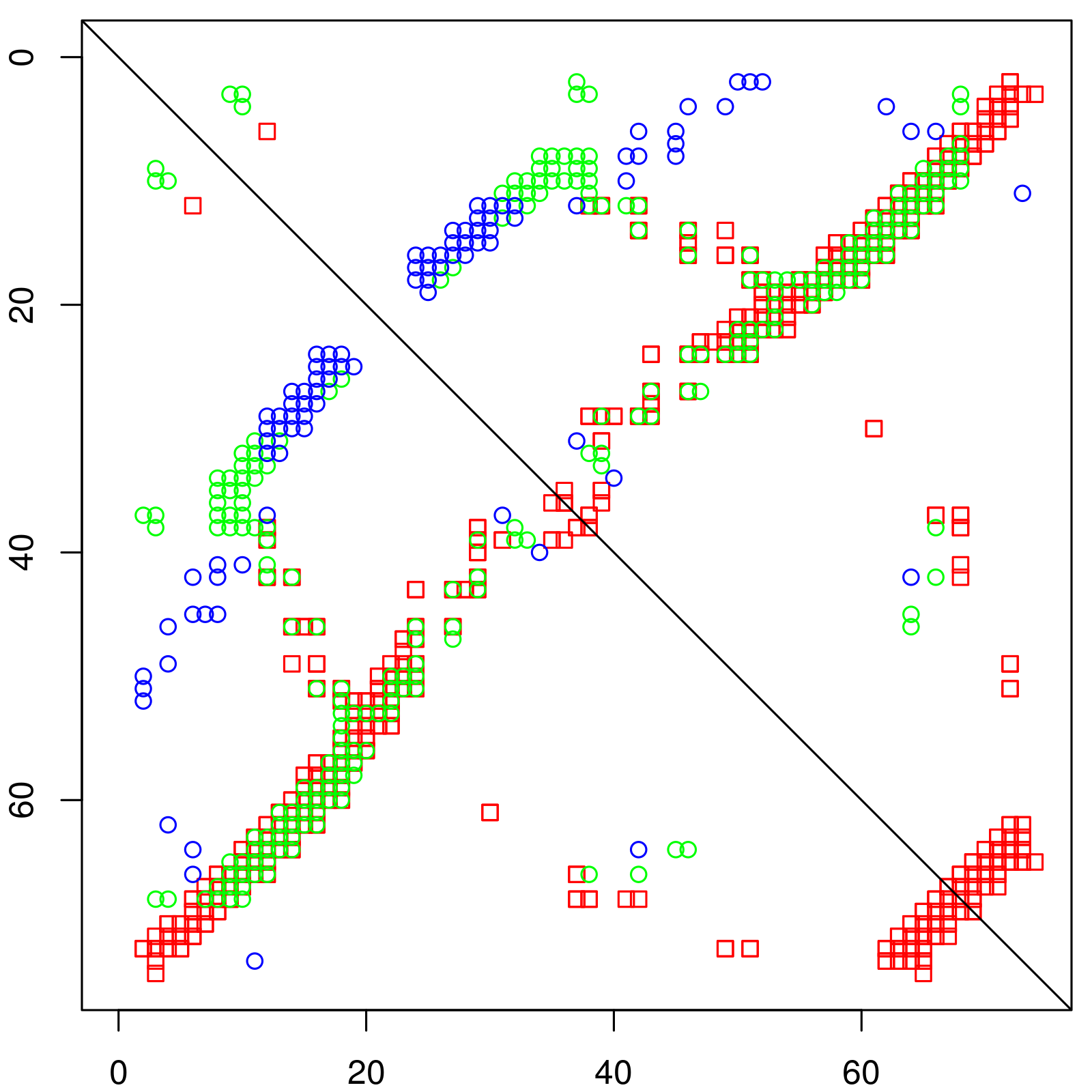(a) No relax removal | 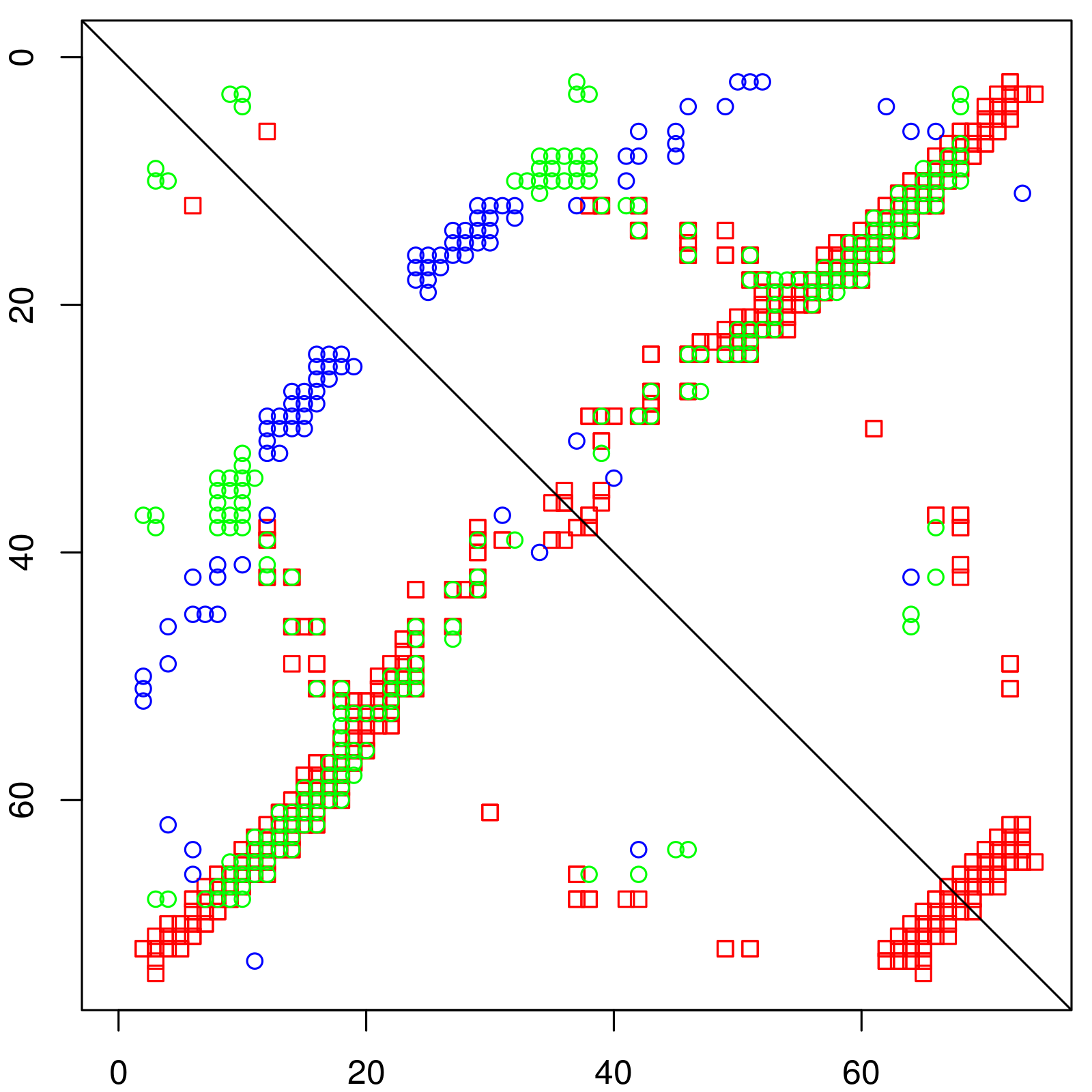(b) Relax removal = 1 | 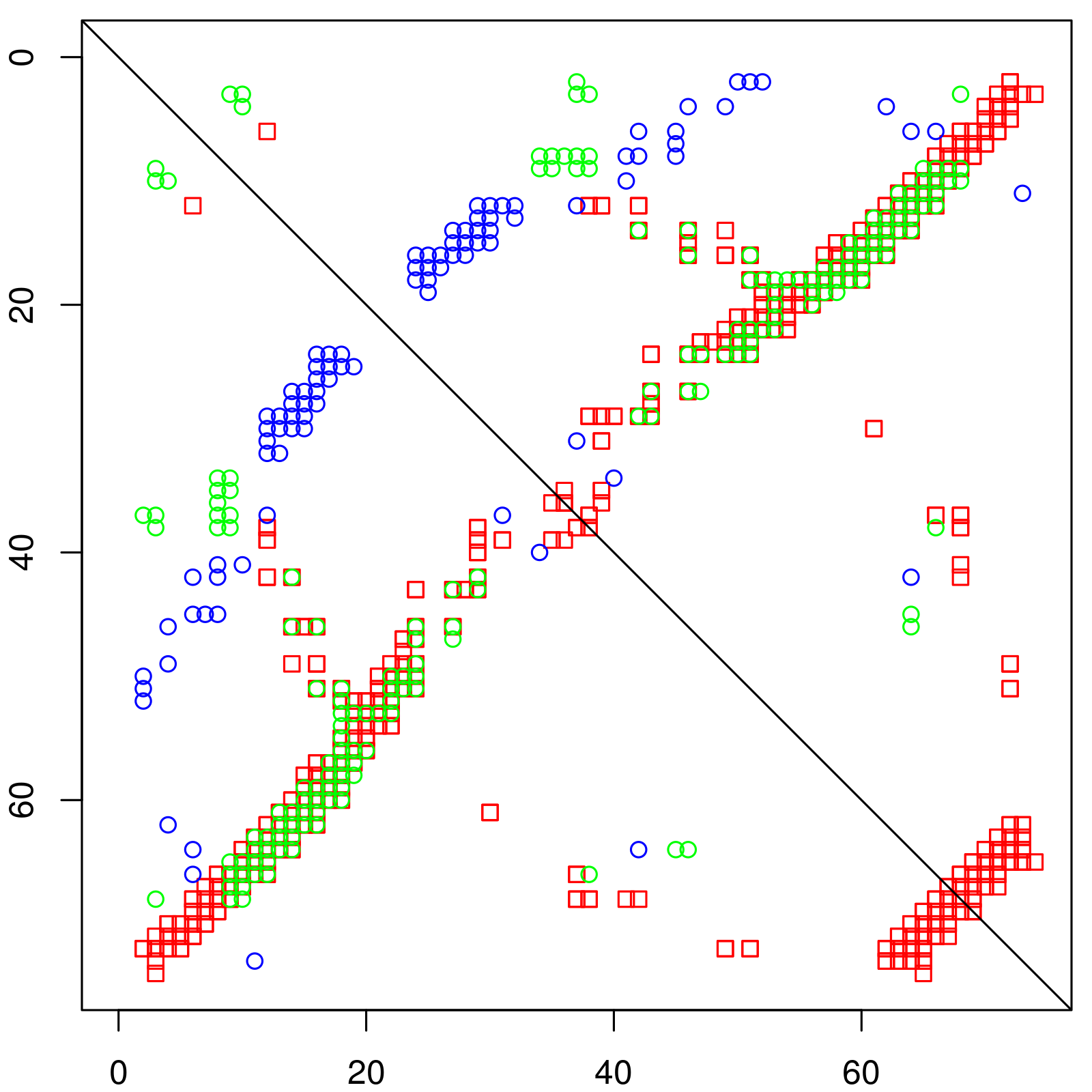(c) Relax removal = 2 |
| --- | --- | --- |

**Figure S5**: Contact map comparison of the Top-L true intrachain (blue), predicted interchain (green), and true interchain (red) contacts for 1IHR for different relaxation removals. (a) shows more green spots overlapping with intrachain blue spots since no relax removal was done. From (b) to (c), the green contacts become sparser due to removing more predicted contacts assumed to be intrachain. According to Table , the final Top-5, Top-10, Top-L/10, Top-L/5, Top-L/2, and Top-L precisions for this prediction are all 100%. Only the Top-2L precision drops to 99.32%, 97.97%, and 89.86% for relax removal 0, 1 and 2, respectively.

| 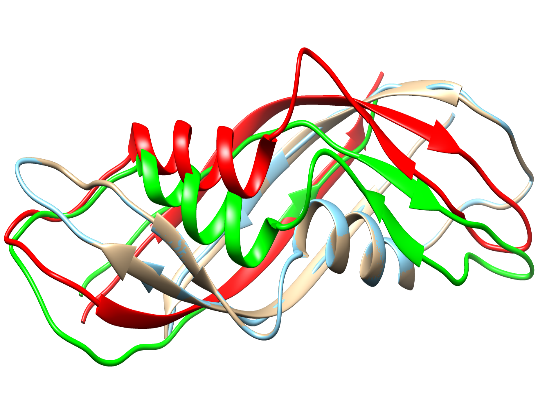  (a) No relax removal | 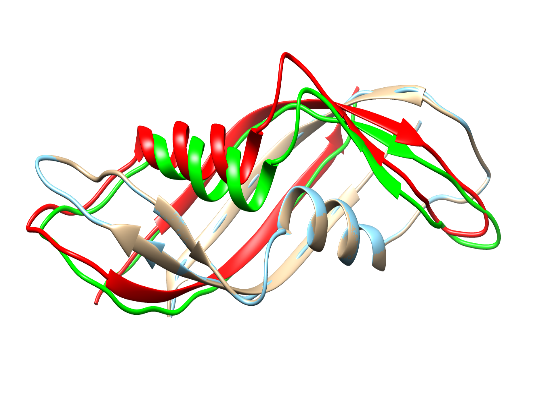  (b) Relax removal = 1 | 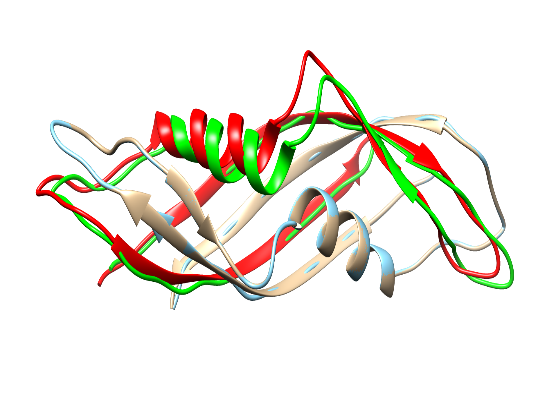  (b) Relax removal = 2 |
| --- | --- | --- |
| \| TM-Score \| 0.79282 \| \| --- \| --- \| \| RMSD \| 2.49 \| \| Length \| 74 \| \| Interchain precision \| 99.32% (2L) \| | \| TM-Score \| 0.90649 \| \| --- \| --- \| \| RMSD \| 1.42 \| \| Length \| 74 \| \| Interchain precision \| 97.97% (2L) \| | \| TM-Score \| 0.91896 \| \| --- \| --- \| \| RMSD \| 1.29 \| \| Length \| 74 \| \| Interchain_precision \| 89.86% (2L) \| |
| 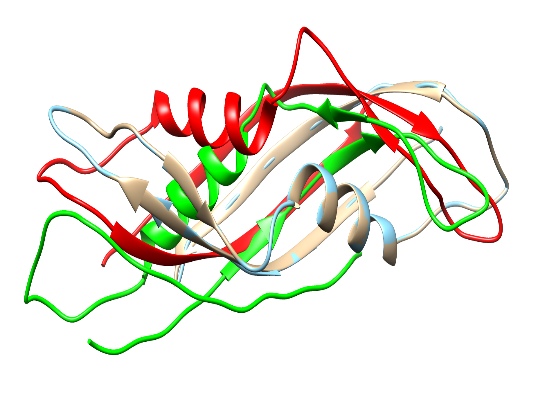  (d) No relax removal | 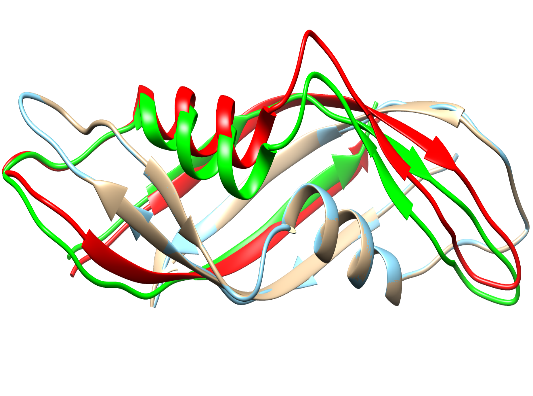  (e) Relax removal = 1 | 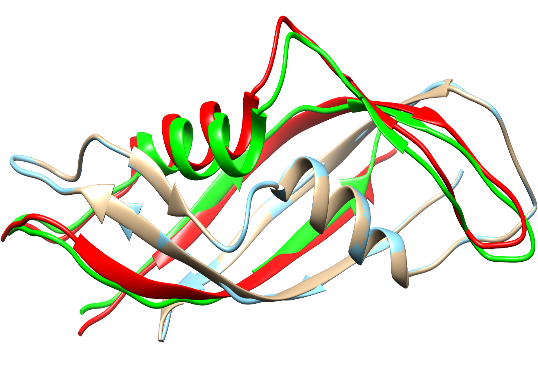  (f) Relax removal = 2 |
| \| TM-Score \| 0.63100 \| \| --- \| --- \| \| RMSD \| 3.99 \| \| Length \| 74 \| \| Interchain precision \| 100% (L) \| | \| TM-Score \| 0.93180 \| \| --- \| --- \| \| RMSD \| 1.19 \| \| Length \| 74 \| \| Interchain precision \| 100% (L) \| | \| TM-Score \| 0.94390 \| \| --- \| --- \| \| RMSD \| 1.04 \| \| Length \| 74 \| \| Interchain precision \| 100% (L) \| |

**Figure S6:** Comparison for target 1IHR between its true homodimer structure (Golden: original chain A; red: original chain B) and the structure derived from predicted contacts (Cyan: reconstructed chain A; green: reconstructed chain B). The TM-score and RMSDs were obtained using TM-Align. From (a) to (c), as we perform relax removal, the TM-score increases while RMSD decreases. But interchain precision (Top-2L) decreases slightly. Structures (d) to (f) are based on Top-L contacts, all of which have 100% precision leading to much higher TM-score and lower RMSD values. The TM-score of (d) was low (even less than (a)) due to the low overlap of the alpha-helix and some noodle regions.

**9.0 The Precision of Intrachain Contact Prediction**

**Table S11:** Table showing the average precision of predicted intrachain contacts obtained for the homodimers and homomultimers using ConEVA. The sequence separation of contacts is short-range or above (>= 6). L represents the length of the protein sequence.

| **Precision (%)** | | | | | | |
| --- | --- | --- | --- | --- | --- | --- |
| **Dataset** | **Top-5** | **Top-L/10** | **Top-L/5** | **Top-L/2** | **Top-L** | **Top-2L** |
| **Dimer** | 96.01 | 94.25 | 92.08 | 85.54 | 74.81 | 57.05 |
| **Multimer** | 89.41 | 87.60 | 85.33 | 79.47 | 70.11 | 54.02 |

**Table** **S11** shows the average precision of intrachain contact predictions for homodimers and homomultimers. Precision is relatively high because both short-range and medium/long-range contacts are considered, and generally good multiple sequence alignments are obtained for the proteins in the dataset. Precision values drop as the number of predicted contacts is increased from Top-5 to Top-2L (L being the protein length). The average intrachain precision for the homomultimers is slightly lower than that of the homodimers.

**10.0 Explanation for Drops in Precision and how Relaxation and relax removal affects precision**

We explain this using **Figure S7** , which shows the frequency of proteins (y-axis) for which a given range of contacts (x-axis) was successfully predicted. Relaxation and relax removal were also varied. The number of successful contact predictions is divided into five categories (x-axis), where 0 means no successful contact was predicted for this protein. A protein is tallied in the range 1-20 if the total number of true-positive predictions for this protein is at least one but less than or equal to 20; and so forth. We show only the Top-L/10 (a) and Top-2L (b) graphs for homodimers in Figure S7 in this analysis for simplicity. A bulk of our samples (especially for Top-L/10) remains mispredicted by DNCON2_Inter (has zero predictions). There are more successful predictions if the number of contacts present in the proteins is within the 1-20 range (Figure S7 (a)). If proteins have more than 20 contacts, the number of successful predictions is low. As we perform relaxation, we can see that the incorrect predictions (zero contact prediction) go down, while successful predictions, especially in the 1-20 range, increases drastically, leading to an increase in precision. As we perform relax removal, the number of proteins in the 1-20 range remains similar, but more well-predicted contacts appear for proteins with more than 20 contacts. This is expected since relax removal removes false-positive contacts from the Top L/10 predictions leading to more true-positive predictions being discovered, thereby increasing precision.

However, we see sharp precision drops in the cases of Top-L/5 and beyond (Figures S7 and S2). We look at the Top-2L graph (Figure S7 (b)) to analyze this observation. Unlike the Top-L/10 graph (Figure S7 (a)), in Top-2L, we see fewer mispredictions (zero value category in x-axis) and more proteins that have been successfully predicted to have 1-100 contacts because 2L is a more expansive range compared to L/10. So more true-positive contacts are encountered. However, during the precision calculation, we are dividing the total number of true-positives by 2L, which is comparatively a higher number. If L is large, the precision drops drastically. As we perform relax removal, we see very little increase in precision for Top-L/5 and beyond, while precision also decreases in some instances (Figures S2 and S7). The Top-2L graph from Figure S7 (b) further suggests that relax removal increases the number of mispredictions (zero value category in the x-axis) while the number of proteins for which successful contacts were predicted decreases. This is due to removing some well-predicted interchain contacts from the predicted contact map when performing the relax removal. In some cases, we discovered that all the predicted contacts get removed, resulting in precisions to become zero.


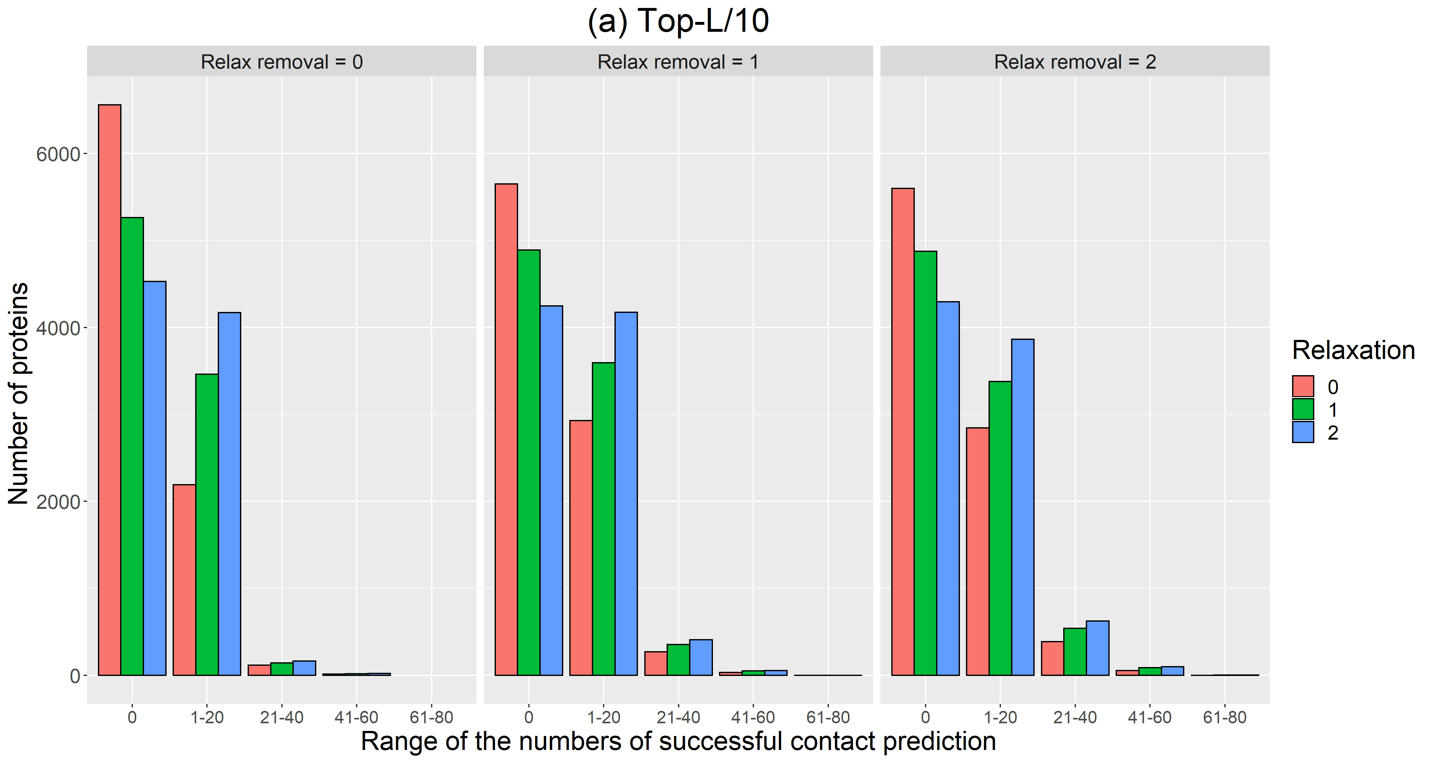


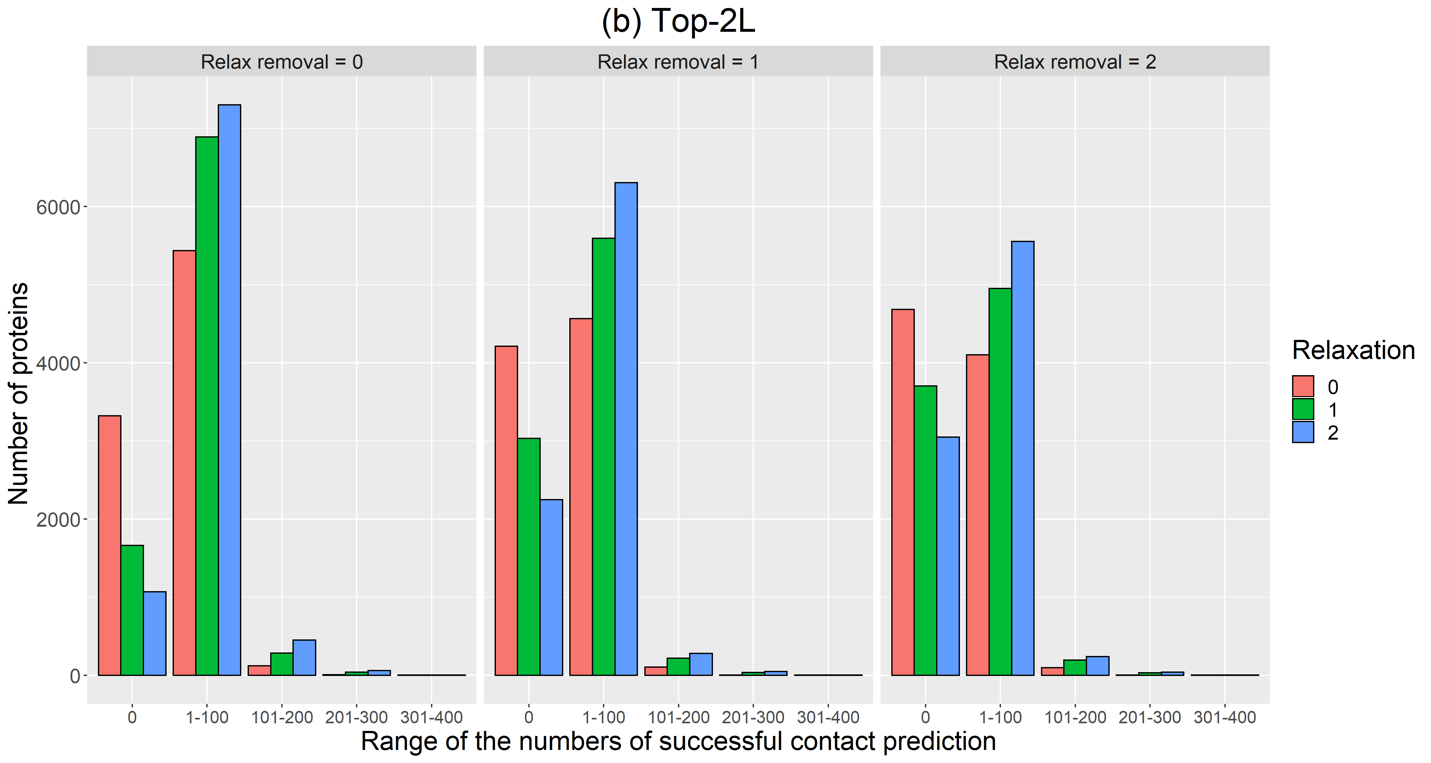


**Figure S7:** The number of homodimeric proteins (y-axis) for which the total number of contacts were successfully predicted within given ranges of total true-positive contacts (x-axis) at different combinations of relaxations and relax removals for (a) Top-L/10 and (b) Top-2L, respectively. (a) The L/10 shows more mispredictions (zero value in x-axis). Among the proteins whose contacts were successfully predicted, most of the proteins have one to 20 true-positive contacts. (b) The bottom graph shows similar results for Top-2L, but most proteins have total contacts within the 1-100 range. In both graphs, we see relaxation increases the number of proteins with well-predicted contacts. Relax removal for the Top-L/10 group increased the number of proteins having more than 20 true-positive contacts. However, for Top-2L, relax removal decreased the number of well-predicted proteins, especially those with one to 100 contacts.

**11.0 How Relax removal, relaxation, and contact density affects precision of DNCON2_Inter and random predictions in homodimers.**








**Figure S8:** Heatmaps showing how the precision is affected by relax removal, relaxation, and contact density ranges for homodimers. (a) Shows the precisions for DNCON2_Inter prediction, and (b) shows the precision changes for random prediction.

**12.0 Brief discussion on how Con_Complex works**

We also selected 1A64 and 1IHR two best-case results and reconstructed their homodimeric complex structure using our recently developed tool Con_Complex, which can reconstruct the quaternary structure of multimers by leveraging the simulated annealing protocol of CNS (Crystallography and NMR System). It uses the monomer PDB file and the predicted inter-protein contacts which are then used as restraints to reconstruct the homomultimeric complex structure. The contact map is designed into five columns such that columns one and two are the residue numbers of the interacting residues of the interface of the respective chains. The third and fourth columns are the distance restraints. The third column is the lower bound of zero Angstroms. The fourth column is the upper bound and, unlike intra-chain contacts, the upper bound distance restrain is set to 6 A for inter-chain contact.  Con_Complex generates 100 intermediate complex structures and then selects the best 5 homomultimer structures based on the lowest cns-energy function. Furthermore, this tool can also handle reconstruction of any other multimers. Preliminary results suggest that the performance of this software heavily depended on the number of good inter-protein contact predictions.
